# Variability in the circulation of cerebrospinal fluid: causes and clinical implications for intraventricular drug delivery

**DOI:** 10.3389/fddev.2026.1735474

**Published:** 2026-05-12

**Authors:** Herbert H. Engelhard, Olivier Balédent, Matthew T. Borzage, Lydia Andrews-Jones, Thomas M. Feldsien, Didier R. Lefebvre

**Affiliations:** 1 AbbVie, Biologics Development (Chemistry, Manufacturing, and Controls), North Chicago, IL, United States; 2 Department of Medical Image Processing, University Hospital, Amiens, France; 3 CHIMERE INSERM UA 21, Universitè de Picardie Jules Verne, Amiens, France; 4 Fetal and Neonatal Institute, Division of Neonatology, Children’s Hospital Los Angeles, University of Southern California, Los Angeles, CA, United States; 5 Alfred E. Mann Department of Biomedical Engineering, Viterbi School of Engineering, University of Southern California, Los Angeles, CA, United States; 6 Department of Regulatory and Quality Sciences, Alfred E. Mann School of Pharmacy and Pharmaceutical Sciences, University of Southern California, Los Angeles, CA, United States; 7 AbbVie, Preclinical Safety, Longmont, CO, United States

**Keywords:** adeno-associated virus, brain, cerebrospinal fluid, choroid plexus, intraventricular drug delivery, Ommaya reservoir, phase-contrast magnetic resonance imaging, subarachnoid space

## Abstract

Understanding the many factors affecting the circulation of cerebrospinal fluid (CSF) is important for the treatment of neurological diseases, especially when considering macromolecular pharmaceuticals designed to be delivered directly into the ventricles of the brain. CSF production, pressure, flow, and absorption are tightly linked to the maintenance of brain homeostasis and function. Vascular pulsations help propel CSF through the ventricular system and subarachnoid space, with additional contributions made locally from the coordinated beating of ependymal cilia. Along with the anatomic configuration of the CSF pathways, these are critical determinants for moving therapeutic molecules and vectors to specific regions of the central nervous system (CNS) and their eventual clearance. The advent of phase contrast MRI has revolutionized the understanding of fluid flow in the human CNS, which is affected by circadian rhythm, age, molecular factors, and diseases, to name but a few. In this review, the basic principles and recent advances critical for understanding the considerable variability of CSF circulation are examined, considering differences that are encountered between healthy individuals and due to disease states. While the emphasis is on human data, illustrative comparisons with laboratory animals are made to enable translation of preclinical data to clinical use. This literature review is intended to be applicable to a wide variety of therapeutic agents, from drugs and macromolecules to adeno-associated virus (AAV) capsids, that are intended for intraventricular delivery.

## Highlights

- Factors affecting the circulation of CSF, and their relative importance, are incompletely understood.
- There is renewed interest in the intricacies of CSF flow, given the development of novel agents for intraventricular delivery.
- We review basic principles and recent advances related to CSF circulation and the implications for intraventricular drug delivery.

## 1 Introduction

### 1.1 CSF flow

As stated by Magram and Liakos 25 years ago, “Although the circulation of cerebrospinal fluid (CSF) enjoys an apparent simplicity, its underlying basis is amazingly complex. Many factors influence how CSF flows through the human central nervous system (CNS)” (Magram and Liakos, 2000). In the 1990s, it was demonstrated that CSF circulation responds to variations in intracranial pressure (ICP) induced by vascular pulsations and respiratory cycles (Enzmann and Pelc, 1993; Greitz, 1993; Schroth and Klose, 1992). Advances in magnetic resonance imaging (MRI) techniques—notably, the development of phase contrast (PC) MRI—has more recently increased the understanding of brain-fluid dynamics (Ren et al., 2024). Each time the heart beats, a traveling wave propagates through the choroid plexus and along CSF pathways which, along with CSF production, propels fluid motion. CSF macrocirculation is defined as the bulk flow or large-scale movement within and between the ventricles and then out into the basal cisterns, subarachnoid space (SAS), and spinal canal. CSF microcirculation involves smaller-scale or localized fluid dynamics, such as currents along the ventricular wall, within brain sulci, and along perivascular pathways (Naseri Kouzehgarani et al., 2021; Iliff et al., 2012). It is now well-established that decreased CSF flow contributes to cognitive decline in the elderly and patients with neurodegenerative diseases (Attier-Zmudka et al., 2019).

A more detailed appreciation of the factors causing variability in CSF circulation (summarized in Table 1) will further enhance the understanding of CNS disease processes, especially those which restrict CSF flow and/or cause accumulation of toxic molecules. Such appreciation will also increase the chance of successful future clinical attempts at drug delivery into the CSF, especially those involving larger molecules, viral vectors, and even cellular therapies (Naseri Kouzehgarani et al., 2021; Priceman et al., 2018). Drugs can be administered directly into the CSF through several routes, including intrathecal injection (such as via lumbar or C1–C2 puncture), intracisternal injection (often used in rodent models), or intraventricular administration (the topic of this review). Therapeutic substances may be delivered in a single dose through multiple doses or by continuous infusion (Naseri Kouzehgarani et al., 2021). For access to the brain or ventricular system, the predominant cranial to caudal CSF flow direction determines that drug delivery via the intraventricular route is superior to intrathecal injection for multiple therapeutic agents (de Oca Delgado et al., 2018; Wilcox et al., 2024; Boyd et al., 2025).

**TABLE 1:** General factors affecting CSF circulation.

CSF production (predominantly at the choroid plexus)	Barrier integrity
Variations in the anatomy of CSF pathways	Physiological bottlenecks
CSF dynamics/bulk flow (pressure gradients)	Intracranial pressure
CSF pulsations/transmission of the waveform	Age
Ciliary motion: ventricular system and central spinal canal	Posture and body movement
CSF clearance/absorption (e.g., venous pressure)	Medications
Frank CSF obstruction due to congenital malformations, hemorrhage, brain tumors, and degenerative changes	Anesthesia
CNS diseases (usually non-obstructive): e.g., infection and inflammation	Intraventricular catheter placement

### 1.2 Intraventricular drug delivery

Interest in investigating the intricacies of CSF flow has recently been revived since sophisticated therapeutic agents have been designed which might be administered directly into the CSF to treat a wide range of neurological diseases (Engelhard et al., 2020; Galvan et al., 2021; Meyer et al., 2023). Currently, the use of intraventricular injection is limited, being confined to specific medical situations such as delivering chemotherapy or antibiotics directly into the brain’s ventricles. Potential advantages of intraventricular delivery for a wider range of indications include enhanced drug delivery by circumventing the blood–brain barrier, higher local drug concentrations at targeted regions and/or disease sites, and reduced systemic side effects. Larger molecules and vectors can potentially be delivered with improved pharmacokinetics than by systemic administration. Advective flow is a process whereby substances within a fluid (such as drugs) are carried along with the bulk movement of the fluid. This flow has at times been loosely termed “convective,” despite the absence of a thermal or density gradient.

Intraventricular gene therapy using viral vectors is currently of particular interest for neurodegenerative conditions, with the intraventricular delivery route having the potential for broad CNS coverage, especially to regions (such as the basal ganglia) that are in close proximity to CSF pathways (Patel et al., 2024). Some non-human primate (NHP) data have indicated that intraventricular injections do not produce CNS coverage as broad as that seen with intracisternal delivery (Ohno et al., 2019). However, injection into the cisterna magna is usually avoided in humans due to the risk of hemorrhage, stroke, or direct medullary injury. As further improvements in understanding CSF pathways and drug design continue to be made, the intraventricular route may ultimately be preferred. Intraventricular drug delivery in animals (called “intracerebroventricular (ICV) delivery”) is technically more difficult than in humans due to the size of the ventricles. A comprehensive understanding of the factors which may be optimized to improve the efficacy of intraventricular delivery in both preclinical work and in patients is highly desirable. A list of therapeutic agents that have been (or could potentially be) injected or infused into the ventricular system is shown in Table 2.

**TABLE 2:** Representative agents that have been used clinically or proposed experimentally for intraventricular therapy.

Category	Example
Drugs	Chemotherapeutics (methotrexate and cytarabine), antibiotics (vancomycin and gentamicin), analgesics, antiepileptics, and corticosteroids
Macromolecules	Monoclonal antibodies, enzymes, recombinant proteins, peptides, cytokines (e.g., interferons), growth factors (e.g., GDNF and BDNF), and immunomodulators
Nucleic acids	Antisense oligonucleotides (nusinersen and tofersen), siRNA, shRNA, plasmid DNA, and mRNA
Viral vectors	Adeno-associated virus, lentivirus, adenovirus, and herpes simplex virus vectors
Cell-based therapies	Neural stem cells, mesenchymal stem cells, genetically engineered cells, and CAR-T cells
Nanoparticles	Liposomes, polymeric nanoparticles, exosomes, and dendrimers
Diagnostic agents	Contrast agents, radiolabeled tracers for PET or SPECT, or paramagnetic or superparamagnetic contrast agents for MRI
Toxins/biologics	Botulinum toxin and other bacterial toxins, such as in combination with targeting agents

Abbreviations: GDNF - Glial cell line-derived neurotrophic factor, BDNF - Brain-derived neurotrophic factor, siRNA - Small interfering RNA, shRNA - Short hairpin RNA, DNA - Deoxyribonucleic acid, mRNA - Messenger RNA, CAR - chimeric antigen receptor, PET - Positron emission tomography, SPECT - Single-photon emission computed tomography, MRI - magnetic resonance imaging. (Naseri Kouzehgarani et al., 2021; Schreiner et al., 2025; Lu et al., 2023; Male and Gromnicova, 2022; Moreira et al., 2024; Ozceylan and Sezgin-Bayindir, 2024; Calinescu et al., 2021; Williams et al., 2025; Nellan et al., 2018; Yue and Shen, 2023; Engelhard et al., 2026; Gutova et al., 2019).

### 1.3 Access to the ventricular system

**FIGURE 1:**
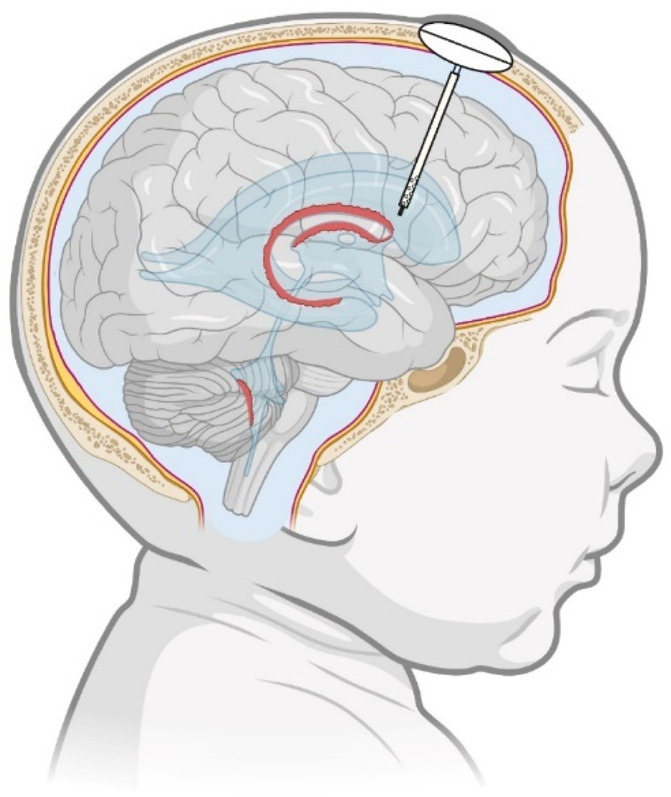
Position for frontal Ommaya burr hole reservoir implantation. CSF pathways are indicated in blue. Choroid plexus is indicated in red. Alterations in catheter position and patency can affect intraventricular drug distribution.

Intraventricular drug delivery requires temporary or longer-term ventricular access and is therefore an invasive procedure. Catheter placement and the migration of fluid around the catheter are significant factors affecting the flow of CSF and administration of therapeutic agents. The two major trajectories used for intraventricular catheter placement in patients are 1) frontal and 2) occipital. While these are mainly used for ventriculoperitoneal shunt placement for hydrocephalus, different catheter positions could influence the intraventricular delivery of drugs to different regions of the brain (e.g., basal ganglia). Intraventricular drug injections via a frontal Ommaya reservoir have been routinely used for the treatment of infections (ventriculitis) or disseminated cancer (leptomeningeal metastases) for many years, and other systems have been described (Naseri Kouzehgarani et al., 2021; Lee G.Y. et al., 2025; Ommaya, 1963; Collins, 1983; Cohen-Pfeffer et al., 2017). An illustration of a frontal Ommaya reservoir implantation is shown in Figure 1. Use of such a reservoir allows repeated dosing or continuous infusion. When using an Ommaya reservoir, the injection of CSF or preservative-free vehicle after drug administration is required to ensure that the drug enters the ventricular system as intended (Tuohy et al., 2025). For long-term (chronic) infusion, a pump and reservoir system can be implanted (Dakhil et al., 1981).

### 1.4 Content of this review

Our aim here is to review recent advances in the understanding of CSF circulation to consolidate knowledge from the published literature. In the following sections, CSF production and the intricacies of the anatomy of the CSF pathways are first examined. The manner in which cardiac and respiratory cycles contribute to CSF currents, superimposed upon the steady flow generated by baseline CSF production, is then described in detail. CSF circulation is subject to alteration by changes in physiological state, anesthesia, medication, and other patient-specific characteristics such as concurrent disease. Examples of patient-specific considerations that may require examination to optimize dosing regimens and reach the therapeutic window (i.e., the doses that achieve efficacy but are less than toxicity) are presented. Two themes will be emphasized: 1) the differences between human findings and animal models and 2) variations that exist between patients and the relative significance of these. The overall goal is to provide information to develop drug delivery strategies, improve therapeutic outcomes, and minimize adverse effects.

## 2 Determinants of CSF production

### 2.1 Basic considerations

Apart from foundational studies of basic anatomy and CSF composition, CSF production is likely the best understood factor affecting the circulation of CSF. CSF is produced mainly by the choroid plexus epithelium within the ventricular system, with approximately 20% produced by other structures including ventricular ependymal cells and via exchange of interstitial fluid (Miyajima and Arai, 2015; Nakada and Kwee, 2019; Liu G. et al., 2022). CSF production needs to be coordinated with CSF resorption (without restriction of CSF flow) or pathological dilation of the ventricles (hydrocephalus) will result. In an adult human, approximately 500 mL of CSF is produced daily into a system where the ventricular capacity is approximately 25 mL and the subarachnoid capacity is approximately 125 mL (Damkier et al., 2013; Sakka et al., 2011). Thus, the total CSF volume is turned over three to four times daily, with the ventricular volume being flushed even more rapidly (Naseri Kouzehgarani et al., 2021). CSF is resorbed via several mechanisms, including resorption via arachnoid villi into venous sinuses and resorption into the lymphatic system via the perineural subarachnoid spaces of cranial nerves (Leinonen et al., 2018). These are discussed at the end of Section 3.

### 2.2 Barriers and cellular interfaces at CSF production sites

The blood–brain barrier is created by tight junctions between capillary endothelial cells in combination with additional restrictions imposed by perivascular pericytes and astrocyte foot processes. The blood–CSF barrier has a different microscopic structure. The capillaries of the choroid plexus are fenestrated, allowing movement of plasma constituents into the stroma of the choroid plexus. The choroid plexus epithelial cells are modified ependymal cells, which are distinct from the ependyma lining the ventricles. The cells of the choroid plexus are cuboidal and possess numerous microvilli. There are fully developed tight junctions between them which act to restrict the movement of plasma constituents into the CSF. Therefore, the choroid plexus epithelial cells create the majority of the blood–CSF barrier. These cells actively secrete water and ions from the blood plasma into the ventricles through a process involving selective filtration and active transport. CSF is normally very low in protein compared to plasma (Spector et al., 2015; Liu R. et al., 2022; Engelhard and Lefebvre, 2026). Approximately 40% of the choroid plexus in humans is located within the fourth ventricle, 25% in each lateral ventricle, and 10% in the third ventricle.

The interface between the ventricles and the brain is comprised of a single layer of multi-ciliated ependymal cells which have adherens and gap junctions, making this point of entry into the brain far more permeable than the blood–brain barrier or glia limitans (Jiménez et al., 2014; Whish et al., 2015; Serra and Simard, 2023). This increased permeability provides a rationale for the intraventricular administration of therapeutic agents. The circumferential organs (median eminence, area postrema, subfornical organ, vascular organ of lamina terminalis, median eminence, neural lobe of pituitary gland, and pineal gland) are highly vascularized, midline, specialized areas at the margins of the ventricular system. Here, the blood–brain barrier is highly permeable. This enables direct communication between peripheral blood, CSF, and specialized cells with crucial sensory and neuroendocrine functions (Kaur and Ling, 2017; Engelhard et al., 2018; Miyata, 2022; Kandel et al., 2021).

### 2.3 CSF production and ICP

CSF production rates in humans, rhesus macaques, and rats are 18–24 mL/h (average: 22.2 mL/h), 4.2–4.8 mL/h, and 0.12–0.24 mL/h, respectively (Johanson et al., 2008; de Lange, 2013). ICP is chiefly related to CSF production at the choroid plexus and resorption at the arachnoid granulations within the venous sinuses. Knowledge of CSF production is important for predicting drug distribution following intraventricular drug administration and for cross-species dose extrapolation (Hammon et al., 2021). Mathematically, Davson’s equation shows how the CSF formation rate (*CSF*_*fr*_) is related to ICP, superior sagittal sinus pressure (*P*_*sss*_), and CSF outflow resistance (*R*_*out*_) (Davson et al., 1987).

$${\text{CSF}}_{fr}=\left(\text{ICP}-P_{sss}\right)/R_{out}.$$

Pathological conditions affecting CSF production and resorption are numerous, including anything that can affect the choroid plexus and/or absorption over the cerebral convexities, such as meningitis, tumor dissemination, and/or hemorrhage. Unfortunately, the choroid plexus will continue to produce CSF even when hydrocephalus is present, leading to further increases in ICP.

### 2.4 Molecular biology

On the molecular level, aquaporins are membrane proteins that are pivotal in regulating the movement of water across CNS barriers and ensuring homeostasis. Aquaporin-1 is predominantly expressed on the apical membrane of choroid plexus epithelial cells and mediates the water transport essential for CSF formation. Aquaporin-4 is mainly found in astrocytic end feet, ependymal cells, and the glia limitans, coordinating water exchange between blood, interstitial fluid, and CSF compartments. Aquaporin-4 is also found in the choroid plexus, especially in the aging brain (Trillo et al., 2019; Szczygielski et al., 2021; Municio et al., 2023; Mack et al., 2024; Deffner et al., 2022). Transient receptor potential, vanilloid 4 (TRPV4) channel, and sodium/glucose co-transporter 2, which are expressed in choroid plexus epithelial and/or ependymal cells, have recently been identified as being important in CSF production (Hochstetler et al., 2020; Sadagopan et al., 2025). Aquaporins (especially aquaporin-4) also play a role in CSF resorption and are modified by hypoxia, oxidative stress, and inflammation (Johanson et al., 2008; Șerban et al., 2025).

### 2.5 Physiological factors affecting CSF production

Multiple factors affect CSF production (Table 3). Many of these are not related to any pathological condition but reflect normal variations between individuals. Preclinical studies have demonstrated reduced CSF production and altered expression of water channel proteins (e.g., Aquaporin 1) in aged mice (Sadanandan et al., 2024; Scarpetta et al., 2023). In humans, choroid plexus volume increases with age, but perfusion decreases, suggesting that age-related changes may reduce CSF production (Alisch et al., 2021; Sun et al., 2024). Experimental data in mice show that young females produce ∼30% more CSF than age-matched males (Liu et al., 2020). Sex differences also exist in choroid plexus volume and cerebral blood flow, with males generally having larger volumes and lower perfusion (Alisch et al., 2021; Zeng et al., 2024). Plasma osmolarity is a lesser-known factor affecting CSF production, with increased osmolarity decreasing production and decreased osmolarity increasing it (Oreskovic and Klarica, 2010).

**TABLE 3:** Factors affecting CSF production.

Age	Neoplasm (choroid plexus papilloma)*
Sex	Inflammatory/Immune state
Posture	Metabolic/Endocrine factors (e.g., androgen excess)*
Arterial CO_2_ tension	Plasma osmolarity
Circadian cycle	Comorbidities/Infection*
Choroid plexus morphology/Hyperplasia*/Blood flow	Genetic factors* (e.g., trisomy 9p)
Protein/Transporter expression	Sympathetic/Parasympathetic tone
Anesthesia	Medications

*Can increase production.

Șerban et al., 2025; Sadanandan et al., 2024; Scarpetta et al., 2023; Alisch et al., 2021; Sun et al., 2024; Liu et al., 2020; Oreskovic and Klarica, 2010; Lolansen et al., 2025; Wang et al., 2025; Zanello et al., 2022; Nimjee et al., 2010; Hashimoto et al., 2023; Duarte et al., 2020; Guo et al., 2017; Henningsen et al., 2022; Swiderski et al., 2012; Wardman et al., 2023; Tariq et al., 2023; Trevisi et al., 2014; Liu, 2009.

Some recent key findings are that posture can affect CSF production—notably, standing upright versus lying down (supine). Wang et al. (2025) used a rotatable MRI system to show that healthy volunteers had changes in choroid plexus position, surface area, volume (approximately 7.5%), and signal intensity in transitioning from lying to standing, which could affect CSF production, circulation, and ICP. Rodent models have demonstrated a more limited effect of posture on CSF production (Khasawneh et al., 2020), which would seem to correspond with their usual horizontal posture and small size. Prolonged postural changes can even affect ultrastructure and gene expression. Weightlessness (“microgravity”), such as in spaceflight, affects CSF circulation in complex ways, resulting in downregulation in CSF production in microgravity that is upregulated upon return to normal gravity (Zanello et al., 2022; Kramer et al., 2015; Krishnamurthy et al., 2021). The effects of the circadian cycle on CSF flow are discussed in more detail in Section 5.2.

## 3 Anatomy of the CSF pathways: blueprint for intraventricular drug delivery

### 3.1 The ventricular system and CSF cisterns

Intraventricular CSF, after originating at the choroid plexuses, flows through the lateral ventricles, the interventricular foramina of Monroe, the third ventricle (which adds production), the cerebral aqueduct (of Sylvius), and then the fourth ventricle (which again adds production). The ependymal lining of the ventricles encloses the CSF, along with drugs, biomolecules, and particles (including nanoparticles and viral vectors) introduced via intraventricular delivery. The ventricular lining creates a boundary that is much less restrictive than the blood–brain barrier (see Section 2.2 above) but does have mechanisms of transport, such as paracellular transport, transcellular transport, and receptor-mediated endocytosis, which are aided by tanycytes and CSF-adjacent neurons (Jiménez et al., 2014; Serra and Simard, 2023; Groh et al., 2024). The coordinated movement of the motile cilia on ependymal cells is important for maintaining the directional flow of CSF within the ventricular system (Wallmeier et al., 2022).

From the fourth ventricle, CSF then enters the cisterna magna though the median aperture of the fourth ventricle (foramen of Magendie) and to the cerebellopontine cisterns through the lateral apertures (foramina of Luschka). In animals, the cisterna magna is utilized for intracisterna magna (ICM) drug delivery via injection or catheter placement. The foramina of Luschka are likely one-way valves (Hoz et al., 2022). For perspective, the human foramen of Magendie is 2–5 mm in diameter, while in rats it is 1–2 mm. The foramina of Luschka are half as big but are still 40,000 times larger than an AAV capsid (Nowak et al., 2023).

### 3.2 The subarachnoid space (SAS)

After CSF has entered the larger cisterns, it distributes itself across a complex three-dimensional network of sulci and smaller cisterns—the subarachnoid space (SAS) —in an extended network of sheets, coves, and channels that bathe the external aspects of the cerebellum, brainstem, diencephalon, cerebral hemispheres, and spinal cord. The SAS is further “buttressed” by membranous pillars called trabeculae that protect CNS tissue from mechanical shock. Determining a “normal” total volume of CSF in the human proves to be more difficult than would be expected but ranges 140–200 mL, with 75%–85% of this being within the SAS (Sakka et al., 2011; Ma et al., 2025; Haines et al., 2024).

The SAS is bordered by the arachnoid mater (just below the paper-like dura mater) on the external surface and the pia mater on the internal surface of the brain and spinal cord. This delicately follows even the smallest contours of the brain’s surface, including extending alongside blood vessels that course into the parenchyma. The pia mater is made up of a monolayer of distinct fibroblast-like cells and specialized connective tissue, providing physical separation between the CSF and parenchyma. The pial layer has scattered circular openings (stomata), which are approximately 1 μm in diameter (40 times the size of an AAV capsid or 100 times the size of an IgG molecule) (Barshes et al., 2005; Li et al., 1996; Betsholtz et al., 2024).

**FIGURE 2:**
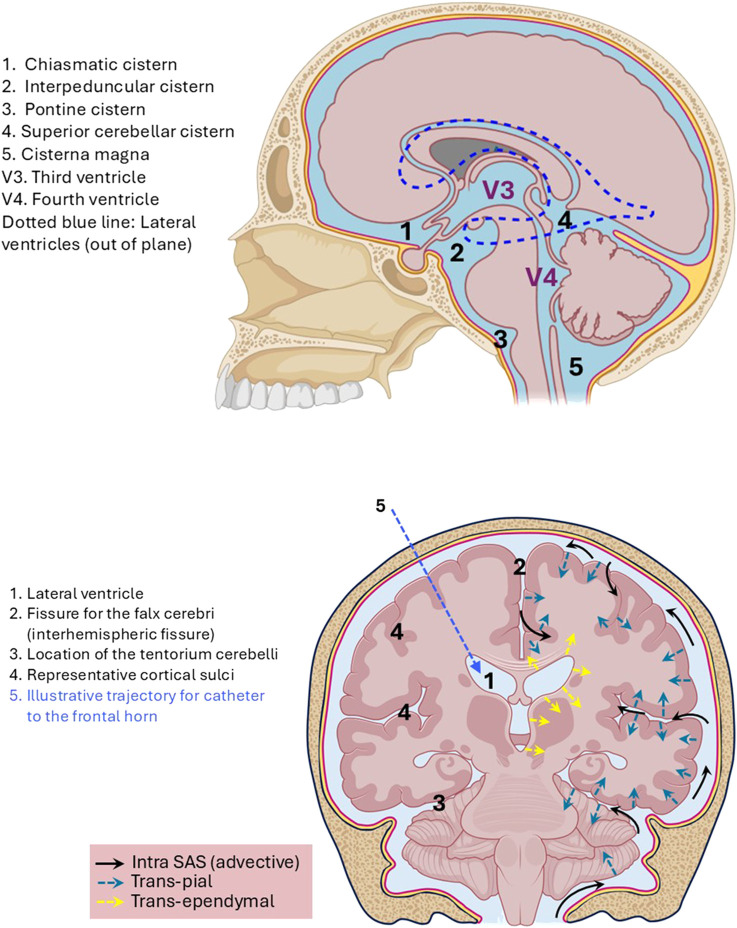
Top panel: midline sagittal section showing the ventricles and major subarachnoid cisterns. The dashed line depicts the overlying position of a lateral ventricle. Bottom panel: coronal brain cross-section with a depiction of three potential transport pathways for CSF-administered drugs. (1) Advective flow (bulk CSF): black arrows. (2) Trans-pial transport (from SAS to cortex and deeper brain tissues): green arrows. (3) Trans-ependymal transport (the ventricles to subcortical tissues): yellow arrows. The blue dashed line indicates a trajectory to the lateral ventricle. Schematic, not to scale.

The volume of the SAS is significantly increased by the presence of the sulci, cisterns, and fissures, which potentially play a significant role in enabling brain tissue exposure to macromolecules and viral vectors administered into the CSF, as they greatly increase available surface area (especially over the cerebral convexities). The cortices are more folded in primates than in rodents (Ventura-Antunes et al., 2013). Major brain structures are labeled in Figure 2, along with potential drug transport pathways. Animals closer to human size (such as pigs and sheep) have also been used to study intraventricular drug delivery (Benatti et al., 2025).

**FIGURE 3:**
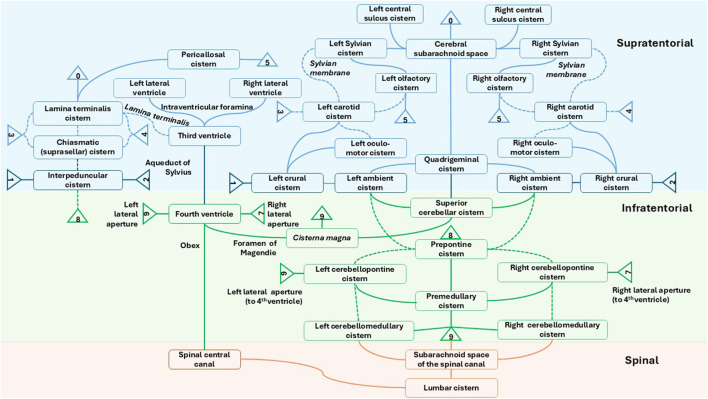
Simplified diagram of the interconnecting CSF-containing ventricles, spaces, and cisterns.

A 2-D projection of the 3-D network of ventricles, “spaces,” and cisterns in humans is shown in Figure 3. The literature does not provide much quantitative information on CSF flow patterns at this level of detail, reflecting the fact that more research and advanced imaging is needed.

### 3.3 Movement of therapeutic molecules in the CSF

Complicating the free flow of therapeutic molecules and particles between cisterns and throughout the SAS are the presence of multiple arachnoid membranes, such as Liliequist’s membrane (which divides the interpeduncular cistern from the chiasmatic cistern) (Zhang et al., 2012; Farooq et al., 2024) and the many membranes (e.g., carotid and Sylvian, but mostly unnamed) that further compartmentalize the cisterns (Kurucz et al., 2013; Rai et al., 2022). These vary in structure, with some forming nearly complete anatomical barriers, while others present as trabeculated networks. Membranes in the carotid membrane group separate the carotid cisterns from the chiasmatic and interpeduncular cisterns. Membranes in the tentorial membrane group separate the ambient cisterns from the quadrigeminal cistern. The clival membrane group separates the prepontine cistern from interpeduncular and pre-medullary cisterns. Membranes in the perimedullary group separate the pre-medullary cistern from the cisterna magna and lateral cerebromedullary cisterns. The number of cisterns is large: approximately 40 in humans. They host traversing blood vessels and cranial nerves. In rodents, it has been demonstrated that gold nanoparticles enter brain parenchyma within 10 min of being injected into the interpeduncular or pontine cisterns (Pan et al., 2023).

Some membranes (e.g., the Sylvian) compartmentalize a single cistern into localized regions. These membranes are not universally present across all individuals—a variability that may have significant implications for drug delivery via CSF (Kurucz et al., 2013). CSF and drugs that have low molecular weight generally flow freely across the cisterns and SAS. Large molecules, such as antibodies and antibody–drug complexes (ADCs), along with viral vectors and nanoparticles, have been reported to flow through the SAS, but their movement is more strongly influenced by CSF flow dynamics, surface adhesion, and arachnoid membranes. Their larger size results in slower diffusion through the CSF (Naseri Kouzehgarani et al., 2021). The transport characteristics of these membranes, including any molecular weight cutoffs for passage of molecules and vectors, need to be identified.

Veins and arteries, ensheathed with a thin membranous layer separating them from the CSF, run in the SAS, forming a tree-like web pressed against the brain, from which depart a multitude of penetrating vessels. Perivascular spaces are thin layers of fluid around these penetrating blood vessels (both arteries and veins) that communicate with the interstitial space of the brain, allowing the transport of solutes, nutrients, hormones, and metabolic products and the clearance of waste products. Perivascular spaces function as part of the glymphatic system, discussed in Section 3.5 below. Around penetrating arteries, CSF enters perivascular spaces via Virchow–Robin spaces. Perivenous channels then allow interstitial fluid and CSF to clear the brain along exiting veins (Iliff et al., 2012; Mehta et al., 2015; Prasuhn et al., 2024; McDonald et al., 2025). All of these cisterns and spaces play potential roles in the transport of drugs, macromolecules, or viral vectors from the SAS to CNS tissues (Naseri Kouzehgarani et al., 2021).

### 3.4 Spinal pathways

**FIGURE 4:**
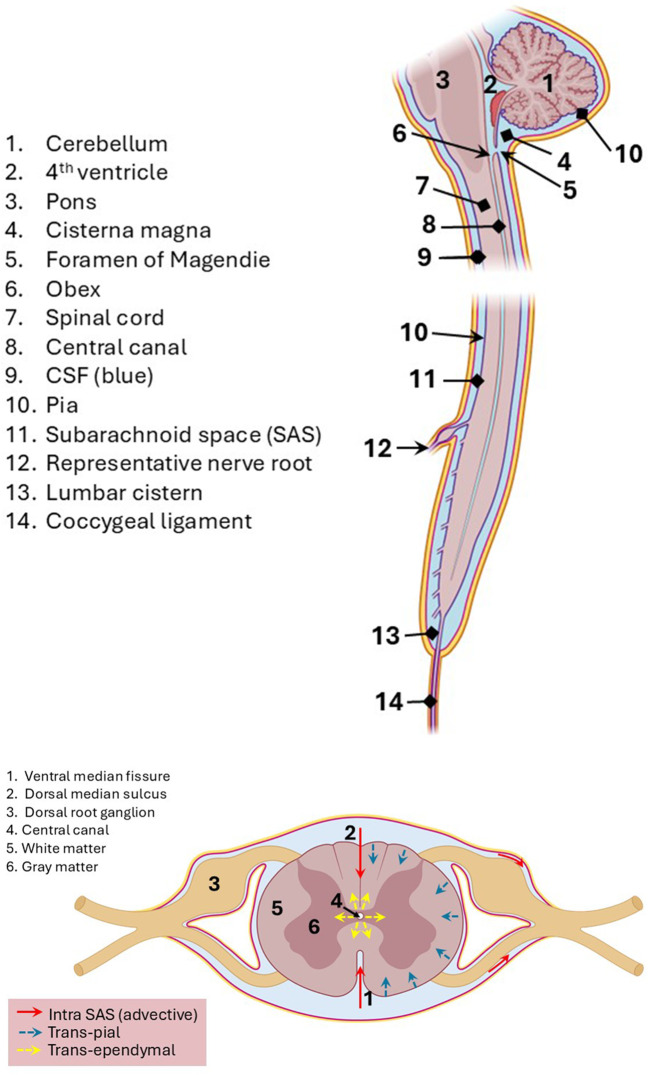
Top panel: CSF circulation around the spinal cord. For simplicity, only one nerve root (with ganglion surrounded by CSF) is depicted. Nerve rootlets emanate from the dorsal and ventral aspects of the spinal cord. CSF flows in and out of the central canal through the obex. Schematic, not to scale. Bottom panel: transverse cross-section of the spine with a description of three potential transport pathways for CSF-administered drugs. Red arrows: advective flow (bulk CSF). Blue arrows: trans-pial transport (from the SAS to spinal tissues). Yellow arrows: trans-ependymal transport (from the central canal to spinal tissues). The ganglia are surrounded by CSF. Schematic, not to scale.

A small fraction of spinal CSF is found within the central canal, a narrow elliptical tube from the fourth ventricle, via the obex, down the length of the spinal cord to the conus medullaris (Figure 4). Like the ventricles, the central canal has an ependymal lining. The central canal, with a diameter of 1 mm or less in humans, is a remnant of the neural tube which guided fetal CNS development. The caudal portion of the central canal is not usually patent in adults. In addition, CSF flows around the spinal cord and for a short distance around the ventral (motor) and dorsal (sensory) nerve roots. A representative nerve root is shown (item number 12) in the top panel of Figure 4. In an anthropomorphic model, the presence of nerve roots has been shown to increase steady streaming in the spinal SAS (Sass et al., 2017; Khani et al., 2018).

Dorsal nerve roots include dorsal root ganglia (DRGs), wherein reside the first-order nerve cell bodies of sensory nerves. These DRG neurons receive sensory information from the periphery and then project this via axons in the dorsal funiculi of the spinal cord to second-order neurons in the gracile or cuneate nuclei in the medulla. Damage to or loss of DRGs can result in lost sensation, pain, or numbness in affected segments. CSF-administered or peripheral-blood-administered viral vectors are known to present a risk of inducing DRG toxicity (Hinderer et al., 2018; Hawley et al., 2025), which tends to be most severe and consistent in the lumbosacral DRGs in non-human primate studies (Buss et al., 2022). Potential transport pathways for CSF-administered therapeutic agents to the spinal cord are illustrated in the bottom panel of Figure 4. The posterior median sulcus and anterior median fissure expand the contact area between the spinal cord tissue and CSF.

After the CSF reaches the lumbar cistern, it circulates within the SAS around the cauda equina, then may move cranially back toward the thoracic and cervical regions. It is not the case that caudal CSF movement is primarily in the dorsal spinal SAS and that cranial movement is in the ventral spinal SAS. There is a difference in the CSF circulatory pattern between the ventral and the dorsal subarachnoid spaces. CSF along the ventral spinal SAS flows in a fairly pulsatile fashion, but flow is more constant in the dorsal spinal SAS (Wachi et al., 1995). The bidirectionality of CSF flow and effect of pulsations (with variations during systole and diastole) are described in Section 4. Intraventricular infusion of gold nanoparticles in mice has demonstrated eventual delivery to the axoplasm of distal peripheral nerves, indicating a contiguous delivery system between the CNS and the peripheral nervous system, a pathway which moves particles up to approximately 15 nm in diameter (Ligocki et al., 2024). Understanding all the intricacies of the anatomical CSF pathways is crucial to predicting successful intraventricular drug delivery to a targeted site within the nervous system as a whole.

### 3.5 CSF clearance

CSF is cleared from the CNS via several pathways. The major outlet is through the cranial arachnoid granulations projecting from the SAS into the dural venous sinuses. These act as miniature valves that help regulate ICP. Some CSF may be cleared via arachnoid granulations within the spinal canal. CSF may also exit through small nerve root sleeves or basal foramina and be absorbed into adjacent blood vessels or lymphatics (i.e., along cranial nerves and spinal nerve roots). To a lesser extent, the meningeal lymphatic network, as well as perineural spaces crossing the roof of the nasal cavity (cribriform plate), also contribute to CSF clearance (Proulx, 2021; Vucevic et al., 2024).

**FIGURE 5:**
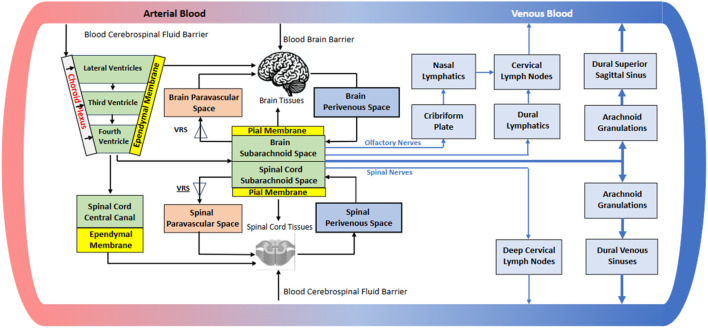
Schematic representation of CSF flow from secretion to clearance. Note: 1) the ependymal membranes lining the ventricles and the ependymal canal; 2) the pial membranes lining the SAS of both the brain and the spinal cord; 3) the Virchow–Robin spaces gating the perivascular spaces. All three boundaries play a role in drug delivery via the CSF. All arrows shown depict CSF flow. The thick blue arrow from the SAS to the dural clearance pathway indicates that it is the primary clearance pathway. Modified from Naseri Kouzehgarani et al. (2021).

More recently, it has also been shown that CSF enters brain parenchyma along subarachnoid periarterial spaces, mixes with interstitial fluid, and is then cleared along perivenous drainage pathways. This circulation has been dubbed the “glymphatic system,” is dependent on aquaporin-4 water channels, and is reportedly more active during sleep (Iliff et al., 2012; Mehta et al., 2015). The glymphatic system, therefore, is a fluid exchange and waste-clearance pathway within the brain parenchyma which links CSF and ISF; it is a component of the overall CSF clearance pathways. Ultimately, most CSF is reabsorbed into the venous circulation. The relative contributions of the different drainage pathways vary according to species, age, and pathology (Agarwal et al., 2024). Impaired CSF clearance has been implicated in neurodegenerative disorders such as Alzheimer’s and Parkinson’s disease (Mehta et al., 2022). CSF clearance pathways are illustrated in Figure 5.

Mathematical modeling predicts that CSF absorption through the arachnoid granulations can increase proportionally by 15% as ICP increases. A simulation indicated that an increase in ICP caused 1) more CSF to exit the SAS through the arachnoid granulations (60%–80%) and 2) less CSF to clear through the cribriform plate (40%–20%) (Vinje et al., 2020). Another study showed a decrease in CSF macrocirculation during a Valsalva maneuver, which causes temporary increases in intrathoracic and intracranial venous pressure and a decrease in cardiac output (Bhadelia et al., 2013).

### 3.6 Comparative anatomy of CSF pathways

**FIGURE 6:**
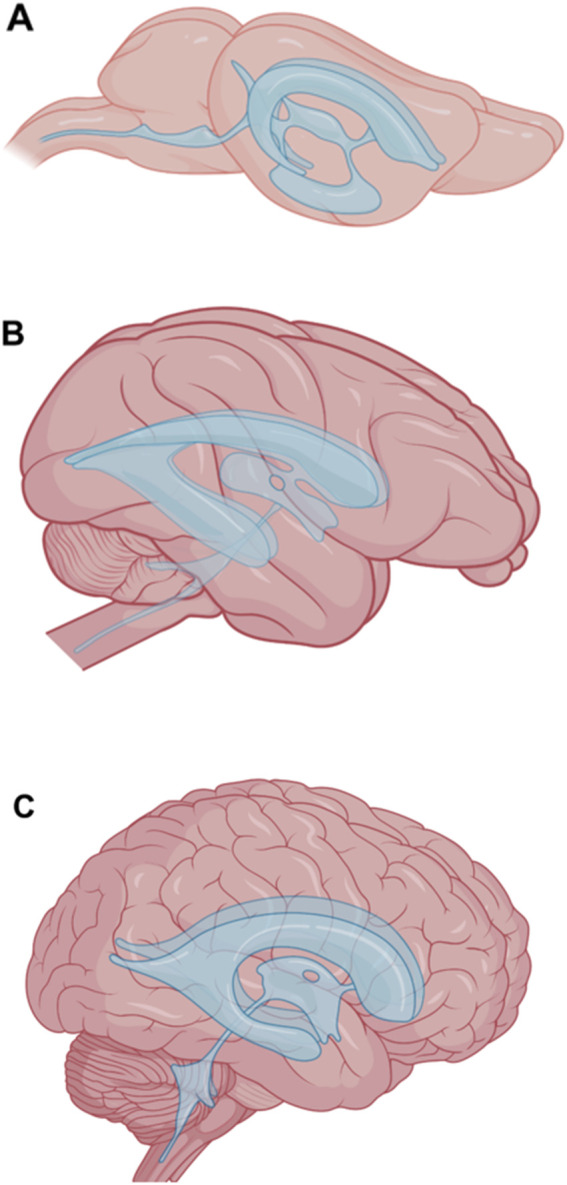
Anatomical differences between the ventricular systems of mice **(A)**, macaques **(B)**, and humans **(C)**. Images are not to scale. The ventricular system is shown in blue. Modified from Schröder et al. (2020).

Relevant to the applicability of animal data to human clinical trials are significant anatomical differences between the CSF pathways of different species beyond just differences in overall size. Rodents have relatively smooth brains, so they will not have the degree of CSF circulation into sulci and crevices (or drug absorption) that humans or NHP will. In addition to their smaller size than humans, ventricles in NHP brains tend to be proportionally narrower. The massa intermedia (intrathalamic adhesion) of a cynomolgus macaque is proportionally much larger than that of humans, reducing their third ventricle to a narrow “ring.” Average values for brain and CSF volume, brain and ventricular surface area, and the surface area to volume ratio for four species is shown in Table 4. Larger animals have lower surface areas relative to their CSF volume, which alters the pharmacokinetics of drugs delivered via the CSF (i.e., drug movement, distribution, concentration, and/or tissue penetration). A major anatomical difference of rodents (and dogs) compared to humans and NHP is the almost 90° turn at the cranio-spinal junction due to the assumption of an upright bipedal posture. The comparative anatomy of the mouse, macaque, and human ventricular systems is illustrated in Figure 6.

**TABLE 4:** Average brain values for four commonly studied species.

Species	Brain volume (cm^3^)	External brain surface area (cm^2^)	CSF volume (cm^3^)	Ventricular surface area (cm^2^)	Total surface area*/Brain volume ratio (cm^−1^)
Mouse	0.3	3	0.04	0.5	11.7
Rat	2.2	20	0.15	1	9.5
Macaque	90	200	15	8	6.8
Human	1400	1850	140	32	1.3

*External brain surface area plus ventricular surface area; values are approximate (Naseri Kouzehgarani et al., 2021; Kandel et al., 2021; Schröder et al., 2020).

### 3.7 Synthesis: significance of pathway anatomy and variations between species

Knowledge of the anatomy and directional flow of CSF pathways is critical in considering what CNS structures will be near drug-delivery routes for therapeutic agents administered into the CSF (Naseri Kouzehgarani et al., 2021; Boyd et al., 2025; Ligocki et al., 2024). The degree of drug movement into the parenchyma after intraventricular administration remains an area of research interest, but the ependymal lining has been shown to be less restrictive than the blood–brain barrier (Jiménez et al., 2014; Whish et al., 2015; Serra and Simard, 2023). Radionuclide imaging can be a powerful tool for characterizing the distribution and kinetics of large molecules such as proteins, antibodies, biologics, and viral vectors administered into the CSF. Radionuclide imaging, fluorescent tracers, and PET studies have shown that, in multiple species, larger molecules administered into the ventricular system move more slowly and/or incompletely into the SAS (Proulx, 2021). Recent human data have shown that a radiolabeled monoclonal antibody administered as a single injection into the ventricles via the Ommaya reservoir is distributed throughout the cranial and spinal SAS within 4 h and then over the cerebral convexity within 24 h (Pandit-Taskar et al., 2019; Kramer et al., 2022). Variations in anatomy can cause differences in CSF flow among individuals within a species as diverse as humans; they certainly impact the ability to extrapolate animal data to humans (Naseri Kouzehgarani et al., 2021).

## 4 CSF flow dynamics

### 4.1 Fundamentals

Cardiac pulsations drive CSF movement, causing alternating bidirectional flow: craniocaudal during systole, and caudocranial during diastole (Causemann et al., 2022; Liu et al., 2025). The pressure fluctuations play a key role in “pumping” CSF into the perivascular spaces and surrounding tissues. The simplified equation for CSF flow, which relates flow (Q_CSF_), pressure difference (ΔP_CSF_), and outflow resistance (R_CSF OUTFLOW_), is

$$Q_{\text{CSF}}=\Delta P_{\text{CSF}}\;/\;R_{\text{CSF}\;\;\text{OUTFLOW}}.$$

**FIGURE 7:**
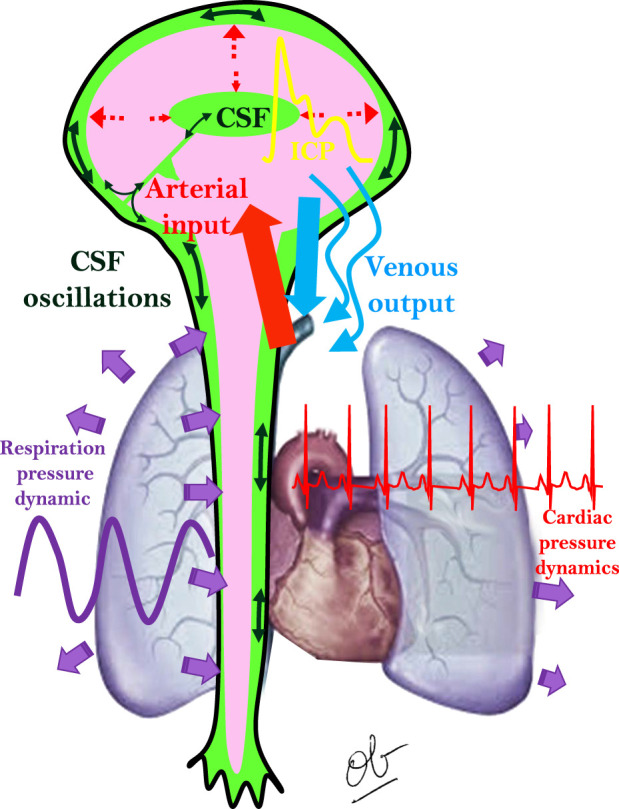
Inter-relationships between CSF flow, arterial input, venous output, cardiac pressure dynamics, and respiratory pressure dynamics. Modified from Balédent (2020).

The pressure difference (ΔP_CSF_) is largely determined by the pulse pressure generated by the cardiac and respiratory cycles. In a modeling approach, other things to consider would include elastance, compliance, and time-varying pressure waves. Stroke volume is the volume of CSF moving back and forth through the upper cervical spinal canal or the cerebral aqueduct during cardiac pulsations (Capel et al., 2014). CSF microcirculation (along the ventricular walls and through Virchow–Robin spaces as examples) is also primarily driven by arterial pulsations (Mestre et al., 2018) and has been likened to peristaltic pumping (Kelley, 2021). Factors that increase the heart rate will increase CSF macrocirculation and microcirculation. The inter-relationships between CSF flow, arterial input, venous output, cardiac pressure dynamics, and respiratory pressure dynamics are illustrated in Figure 7. Outflow resistance, mainly any flow limiting portions of CSF pathway anatomy (mentioned above) and/or increased venous pressure, are important determinants of Q_CSF_.

### 4.2 The impact of phase-contrast MRI in understanding the dynamics of CSF flow

The development of phase-contrast MRI (PC-MRI) has led to breakthroughs in the understanding of cerebral blood flow and the dynamics of CSF flow in humans. Before PC-MRI, CSF was considered to be a relatively stagnant fluid surrounding the brain and spinal cord. Historical aspects of the contribution of PC-MRI in contributing to the understanding of CSF flow are provided in Supplementary Material 1. PC-MRI tracks phase shifts in the MR signal, using a pair of equal and opposite magnetic gradients. In stationary tissues, the gradient effects are canceled, but in moving tissues, the gradients produce a phase shift proportional to velocity. Selecting the strength of the gradient determines the range of velocities encoded (VENC). Cine PC-MRI enables the visualization and measurement of the movement and flow of fluids such as CSF. It works by having the MRI detect the movement of protons in a specific direction. Approximately 30 time points are acquired per cardiac cycle—the signal acquisition rate. CSF moves slowly compared to arterial flow at approximately 5 cm/sec with peaks of nearly 80 cm/sec. Dynamic (velocity curve) images are created by repeating the acquisition over multiple cardiac or respiratory cycles and synchronizing the images to an ECG and respiratory rate monitor.

**FIGURE 8:**
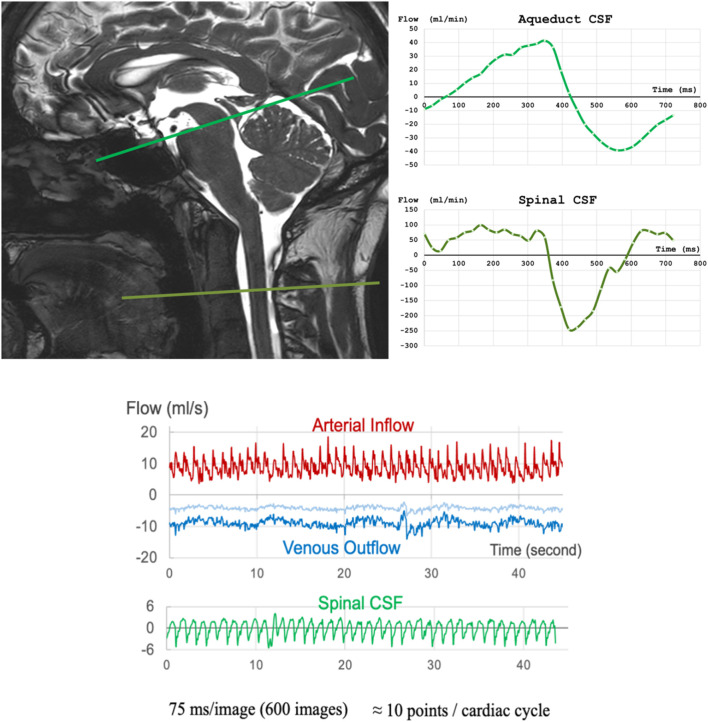
Top: (left) slice positions for CSF oscillation determinations; (right) waveforms indicating CSF flow at the two locations. Bottom: relationship between intracranial arterial inflow, intracranial venous outflow, and spinal CSF flow rates over 45 s.

CSF flow in and out of the spinal canal is measured relative to a slice at the foramen magnum, or just below C2–C3. Due to low internal flow resistance, the intracranial SAS contains the largest CSF volume able to move rapidly through to the spinal canal. Slices taken at the pontine cistern and cisterna magna can simultaneously serve to characterize bulk CSF oscillations and CSF flow through the foramina of Magendie and Luschka. Such foramina could be considered chokepoints of CSF circulation. These slice locations and measured CSF flows at these levels are illustrated in Figure 8 (top panels).

CSF oscillations, primarily driven by cerebral arterial flow, rapidly increase ICP during cardiac systole. In accordance with the Monro-Kellie doctrine (stipulating that the incompressible intracranial volume must remain constant) and given that venous outflow is dampened by resistance, intracranial CSF flows outwardly from the skull into the more compliant spinal compartment. During the cardiac cycle, CSF from the SAS is the first to flow, followed by ventricular CSF (Balédent et al., 2006). During the systolic phase, cardiac pressure forces the sudden volumetric expansion of intracranial arteries, which is compensated by CSF outflow in tandem with intracranial blood outflow through sinuses and veins. The dynamics of CSF flow are also influenced by respiratory movements, which affect intrathoracic pressure and intracranial venous pressure (Yildiz et al., 2017). These relationships, as occurring over 45 s, are plotted in Figure 8 (bottom panel).

**FIGURE 9:**
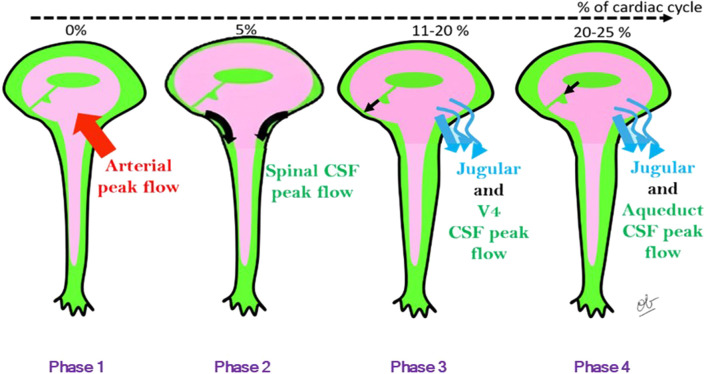
Four phases of cardiac systole, based on CSF and blood peak flows. Phase 1: peak vertebral and internal carotid systolic flow and increased cerebral blood volume. Phase 2: peak CSF flush flow. Phase 3: peak jugular blood flow. Phase 4: peak flush flows at the cerebral aqueduct, third ventricle, and foramen of Monro. Brain CSF filling follows after phase 4. Black arrows represent CSF flow.

Cardiac systole can be divided into four phases, depending on blood and CSF peak flows. In phase 1, there is peak vertebral and internal carotid systolic flow and increased cerebral blood volume. Extracerebral CSF is the first to respond and regulates brain volume expansion by flushing into the spinal SAS. The peak CSF flush flows during phase 2 and at C2–C3 has been measured at 173 mL/min. The elasticity of the dural sac allows the brain CSF flush to be accommodated. Intracerebral CSF at the fourth ventricular outflow level constitutes the second component of brain volume regulation, with an intracerebral CSF peak flush flow of 8 mL/min—much smaller than the cervical peak flush flow. Peak jugular blood flow occurs during phase 3. Peak flush flows at the cerebral aqueduct, third ventricle, and foramen of Monro appear later in phase 4, after the cervical flush. Brain CSF filling then follows. The four phases are illustrated in Figure 9. CSF oscillations can be affected by interventions which modulate cardiac and breathing amplitude and frequency and by venous outflow and pressure.

### 4.3 Cilia and CSF flow

In determining CSF flow, the flux driven by choroid plexus CSF production and vascular pulsations is complemented at a cellular level at the ventricular wall. The surfaces of the ependymal cells lining the ventricles of the brain possess cilia, which beat in a coordinated fashion. The cilia regulate and contribute to CSF macrocirculation (<5%) but mainly affect CSF microcirculation in the ventricles and spinal canal (Olstad et al., 2019; Faubel et al., 2016; Siyahhan et al., 2014; Thouvenin et al., 2020). Ciliary movement helps distribute CSF across the ependymal surface, aiding in its local mixing, homeostasis, and directional guidance. In the central canal of the spine, CSF circulation would be negligible if it were not for the synchronized movement of cilia (Mellanson et al., 2025). The morphology of the ependymal cilia changes with aging (Monaco et al., 2018), and this could influence the CSF microcirculation. The roles that cilia and CSF microcirculation play in aging and neurodegenerative disease are becoming clearer. For example, in rat brain cultures, amyloid beta decreases cilia beat frequency when it would normally be high (during the rest = light cycle) and increases beat frequency when it should be low (during the active = dark cycle). The circadian cycle is discussed in more detail in Section 5.2 below. Amyloid beta has been found to be less neurotoxic when the experiment simulated restored cilia flow (Makibatake et al., 2023).

### 4.4 Synthesis: CSF flow dynamics and intraventricular drug delivery

Of the factors mentioned above, CSF production (Section 2), arterial pulsation, and venous pressure are the most likely to affect CSF macrocirculation and thus the distribution of therapeutic molecules delivered into cranial CSF pathways. Increased CSF flow will increase ventricular flushing and decrease drug contact time with the walls of the ventricle. This concept is important in considering the dosage used for intraventricular drug delivery. On the other hand, factors that affect the CSF microcirculation—such as the integrity of the ciliary motion—affect local CSF and flow mixing and drug or particle adherence to the wall of the ventricle. This is a critical first step prior to penetration into brain parenchyma—a process in which some viruses (such as AAV4) are particularly adept (Naseri Kouzehgarani et al., 2021; Liu et al., 2005; Yamazaki et al., 2014).

## 5 Additional factors in physiological CSF flow variability

### 5.1 Rationale for study

**FIGURE 10:**
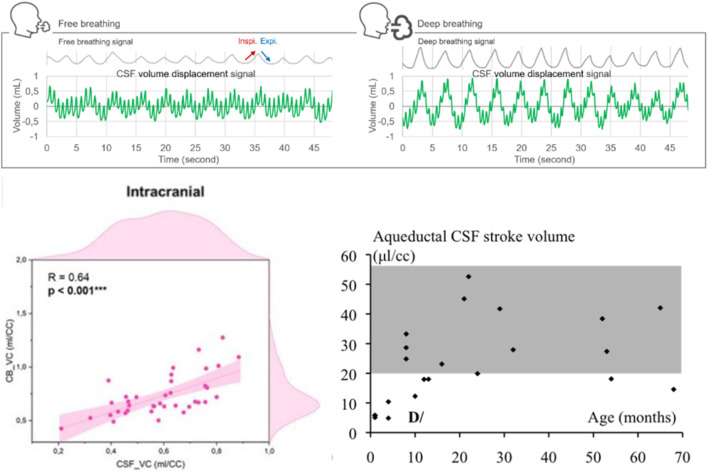
Variations in CSF flow in normal patient populations. Top: effect of respiration and cardiac cycle on CSF flow. Gray: breathing signals were recorded by outfitting the volunteer with a belt sensor. Left: free breathing. Right: deep breathing. Green: plots of CSF volume moving up and down a high cervical section. Note the relative contributions of the respiratory cycles (lowest frequency) and cardiac cycles (highest frequency). Deep breathing notably increases the amplitude of CSF flow across the cervical area. Bottom left: correlation between the stroke volumes of the spinal CSF (CSF_VC) and intracranial cerebral blood (CB_VC) during the cardiac cycle in a population of young adult subjects, illustrating physiological CSF flow variability. Bottom right: cervical and aqueductal CSF stroke volumes as a function of age in children. The gray fields highlight pronounced stroke volume variability [Adapted from Liu et al. (2025), Capel et al. (2014)].

Physiological factors other than variations in anatomy, heart rate, blood pressure, normal respiration, venous pressure, and cilia movement also affect the macro- and micro-circulation of CSF and can result in differences between healthy subjects. These include timing within the circadian cycle, sleep, posture, movement, and age. Consideration of these may help predict the intra- and inter-subject variability of the intraventricular delivery of drugs, viral vectors, and other biopharmaceuticals and further inform the design of clinical trials. Liu et al. (2024) showed that even depth of breathing affects CSF volume displacement (Figure 10, top panel). PC-MRI studies designed to assess the differences in CSF stroke volume in populations of healthy individuals—such as in relation to cerebral blood flow—are seen in Figure 10, bottom left panel. Such data document the heterogeneity seen among normal subjects (Owashi et al., 2024). The wide variation in aqueductal stroke volume seen in healthy children (age 0–70 months) is shown in Figure 10, bottom right panel.

### 5.2 Effects of circadian cycles on CSF flow

Day–night and wake–sleep cycles affect CSF microcirculation and overall flow. In both humans and rats, the dark phase of the circadian cycle increases CSF production 12%–20%, thereby increasing ICP by 30%. In rats, this appears to be driven by changes in a choroid plexus ion co-transporter (NKCC1) and is independent of changes in blood pressure and posture (Steffensen et al., 2023). The increase in CSF production is observed during the human rest cycle (dark phase) but also during the rat’s active night cycle (dark phase), suggesting a conserved ancestral circadian pattern, which remains despite the evolution of different active versus rest patterns. In rats, the expression of a large number of choroid plexus transporter genes that regulate CSF production has been found to differ in the light and dark phases (Edelbo et al., 2023). Light and dark phases even affect the constituents of CSF. In rats, the light phase showed significant increases in inosine, allopurinol, urocanic acid, and melatonin levels, whereas the dark phase showed elevated cytosine and 5-methylcytosine levels. These effects might result from metabolic changes in the brain and not just at the choroid plexus (Edelbo et al., 2023).

In mice, the light (rest) phase of the circadian rhythm is associated with increased CSF microcirculation and perivascular CSF clearance of fluorescent tracers, driven by aquaporin-4 (AQP4) (Hablitz et al., 2020). A rat study injected ferumoxytol tracer into the right lateral cerebroventricular cistern and observed redistribution by MRI over 2 h. There was higher redistribution in the light (rest) phase and in the dorsal cerebrum, and lower redistribution in the dark (active) phase and in the midbrain and periventricular parenchyma (Cai et al., 2020). An MRI study of humans reported that both sleeping after sleep deprivation as well as deeper sleep increased CSF pulsations, which would be expected to increase CSF clearance (Helakari et al., 2023). Natural sleep has been found to increase CSF velocities and low frequency CSF flow oscillations (Fultz et al., 2019). The distribution of a drug administered via the intraventricular route will therefore vary based on the circadian rhythm due to differences in the amount of CSF produced, circulating, and absorbed, depending on the time of day. Intraventricular drug delivery, species-specific differences, and the timing of drug delivery could be important factors in choosing an appropriate animal model and human dosage schedule, since CSF flow can be affected by approximately 20% by the diurnal pattern (Steffensen et al., 2023).

### 5.3 Effects of posture and musculosketetal movement on CSF flow

The hydrostatic pressure of liquids varies with the relative position of the fluid column. When a subject is lying horizontally, hydrostatic CSF pressure is uniform from the head to the bottom of the spinal canal. In the standing position, the bottom of the spinal canal experiences maximum hydrostatic pressure (e.g., 30 cm H_2_O). Likewise, cerebral blood drainage differs between horizontal and vertical positions. In the standing position, peripheral veins play the primary role in venous return, while the internal jugular veins assume a secondary role. This order reverses when the individual lies down (Jurjević et al., 2024; Alperin et al., 2005a; Alperin et al., 2015; Alperin et al., 2021; Gergelé and Manet, 2021). The effect of gravity, or its absence, on cranial–spinal dynamics has become a significant area of interest in the context of human spaceflight (Kramer et al., 2020; Roberts et al., 2017).

Handgrip, head-nodding, locomotion, and Valsalva maneuvers (as well as spasticity) have different effects on CSF flow. Rhythmic handgrip activity was found to decrease CSF macrocirculation (both flow rate and stroke volume) despite increases in respiration and heart rate. This may indicate that it elevates cerebrovascular resistance (mean arterial pressure divided by arterial blood flow) and thereby decreases both vascular and CSF stroke volume (Tarumi et al., 2021). Head-nodding has been found to decrease CSF macrocirculation and increase spinal CSF pressure (Xu et al., 2021). Sitting upright also decreases CSF macrocirculation, likely from gravitational effects causing a decrease in intracranial blood volume from decreased hydrostatic pressure (Alperin et al., 2005b).

### 5.4 Effects of age and sex on CSF flow

From the first days of life to its final stages, the cranial–spinal compartment undergoes gradual changes that affect anatomy and the flow dynamics of the fluids of the CNS. In newborns and children, the dynamics of CSF stroke volume change rapidly with age, primarily due to skull growth (Capel et al., 2014; Borzage et al., 2014). A more detailed consideration of CSF flow dynamics in the pediatric population is provided in Supplementary Material 2. CSF flow is more pronounced in the cerebral aqueduct of an elderly population than a younger one (Lokossou et al., 2020; Vikner et al., 2024). Independent of age or height, CSF dynamics are more pronounced in men than women (Schmid Daners et al., 2012). Non-pediatric clinical trials typically include male and female subjects ranging in age from 18 to 65 years, with the same drug dose and schedule being used across patients. In the case of intraventricular therapies, dosing considerations may need to include factors such as the patency of the pathways and estimated ventricular volume (at a minimum), much as a pediatric dosing schedule is adjusted according to weight.

### 5.5 Synthesis: relative importance of factors producing intersubject heterogeneity and affecting intraventricular drug delivery

Fundamental physiological parameters such as heart rate, blood pressure, and respiratory rate are affected by sympathetic and parasympathetic stimulation, all of which in turn affect blood flow within the choroid plexus. Respiration in turn affects arterial CO_2_ tension (pCO_2_) and cerebral blood flow. Multiple other factors that affect CSF production have been described in Section 2. Physiological factors can be interconnected, such as increased sympathetic tone, heart rate, and choroid plexus blood flow. Standard medical care would naturally entail avoiding abnormalities of blood pressure, heart rate, respiratory rate, pCO_2_, and serum osmolarity.

According to a synthesis of the currently available literature, the most significant factors expected to cause variability of CSF macrocirculation would be overall CSF production, anatomical pathway variation, and age. These would be the same variables that would be expected to have the most impact on intraventricular drug delivery. A comparison of the effects of physiological factors on CSF flow and drug delivery is shown in Table 5. Some of the more nuanced considerations listed below (such as ciliary function) are projected here to have lower overall importance for intraventricular drug delivery and may primarily affect CSF microcirculation. Yet even if judged to have lower importance, the effect on drug binding to the ventricular wall (and subsequent distribution into the parenchyma) could still be significant.

**TABLE 5:** Effect of physiological factors on CSF flow and intraventricular drug delivery.

Physiologic factor	CSF effect	Drug delivery implication	Potential importance
Anatomical variation	Narrowing of pathways may decrease flow	May restrict drug distribution	High
Age	CSF production decreases with age; ventricular system and SAS also relatively larger	Should definitely be a strong consideration	High
Arterial blood pressure	Markedly high pressure can increase CSF flow	High pressure→ increase ventricular flushing	Intermediate
Choroid plexus blood flow	Increased blood flow increases CSF production	Increase → increase ventricular flushing	Intermediate
Heart rate	Affects frequency of CSF pulse wave	Increased pulse may increase propulsion	Intermediate
Venous pressure	Increased pressure impairs absorption	Could affect CSF dynamics—should be considered	Intermediate
Posture and motion	Lying down can raise ICP but also venous pressure	Variable—should be considered	Intermediate
Respiratory rate	May affect CSF waveform and arterial CO_2_ tension.	Variable	Intermediate–low
Arterial CO_2_ tension	Hypercapnia increases production; hypocapnia decreases production	May affect ventricular flushing	Low
Plasma osmolarity	Increased osmolarity decreases CSF production; decreased osmolarity increases CSF production	Modulates ventricular flushing	Low
Sympathetic stimulation	Reduces CSF production	Decreases ventricular flushing	Low
Parasympathetic stimulation	Can increase CSF production	Increases ventricular flushing	Low
Circadian cycle	Can affect CSF production and circulation	Time of day can affect CSF circulation	Low
Ciliary function	Influences microcirculation at ventricular wall	May affect ependymal binding and absorption	Low

## 6 Diseases affecting CSF flow and implications for dosing and patient care

### 6.1 Obstruction of CSF flow

The free flow of intracranial CSF into the spinal canal is fundamental to the homeostasis of the CNS. Diseases directly impairing CSF flow can cause partial or complete obstruction anywhere along the anatomical CSF pathways. Obstructive hydrocephalus, typically associated with major ventricular dilation, is associated with the highest restriction of CSF dynamics and can have multiple underlying causes such as tumors, infections, hemorrhages, trauma, or even ciliopathies—just to name a few. Hydrocephalus may be congenital or acquired, communicating or non-communicating, and presents across the age spectrum. Intraventricular CSF and SAS CSF pulsatility may become decoupled in disease states. Aqueductal obstruction results in hydrocephalus and prevents CSF oscillations between the ventricles and other CSF compartments (Stoquart-El Sankari et al., 2009).

In contrast, normal pressure hydrocephalus (NPH) presents a wide range of dynamic CSF flows, most often with cerebral aqueduct pulsatility levels over ten times higher than normal, while pulsatility in the spinal canal is normal to lower-than-normal (Whitley et al., 2024; Capel et al., 2023a; Stöcklein et al., 2022; Saliou et al., 2012). Hydrocephalus is observed in some cases of dwarfism where patients present a narrow foramen magnum, causing increased flow resistance and altered intracranial compliance (Bosemani et al., 2015). In rare cases, hydrocephalus may be caused by a decrease in cerebral venous outflow caused by a narrowing of jugular veins or sinus thrombosis, which causes intracranial hypertension and ventricular dilatation (Cinalli et al., 2024). Disorders of cilia are rare causes of hydrocephalus, are usually congenital, and may overlap with other syndromic disorders (Sakamoto et al., 2021; Wallmeier et al., 2022). Therefore, the pathologic mechanisms altering CSF flow or restricting its absorption can occur anywhere along the CSF circulation pathways, and frank obstruction and/or ventricular dilatation may or may not occur.

### 6.2 Disease-related alterations in CSF flow without frank hydrocephalus

A precondition to considering intraventricular drug administration is patency—that is, access to the portion of the CNS pathways that contain or are adjacent to the target pathology. Patients with aqueductal stenosis (as a simple example) would not be candidates for intraventricular viral vector therapy for a spinal cord disease. However, diverse pathologies (or comorbidities) may lead to more subtle (than hydrocephalus) alterations in CSF production, the anatomy of CSF compartments, and flow of CSF. CSF flow may be altered with diseases affecting cardiac and/or respiratory dynamics (Lee VK. et al., 2025; Jeon et al., 2024; Agarwal et al., 2018; ElSankari et al., 2012; Imaizumi et al., 2022; El et al., 2011). The flow of CSF at the base of the skull can be altered in cases of Type I Chiari malformation where cerebellar tonsils extend into the foramen magnum. The rigid skull uses this opening to balance systolic vascular expansion by driving the intracranial CSF into the spinal canal. Obstruction of the foramen magnum by the cerebellar tonsils readily affects pulsations of the CSF and intracranial compliance (Alperin et al., 2005c; Alperin et al., 2014; Bhadelia et al., 2023; Capel et al., 2023b). When intraventricular chemotherapy is used for leptomeningeal metastases, MRI or radiotracer studies may be utilized to confirm patency (Wilcox et al., 2024; Engelhard, 2026).

**FIGURE 11:**
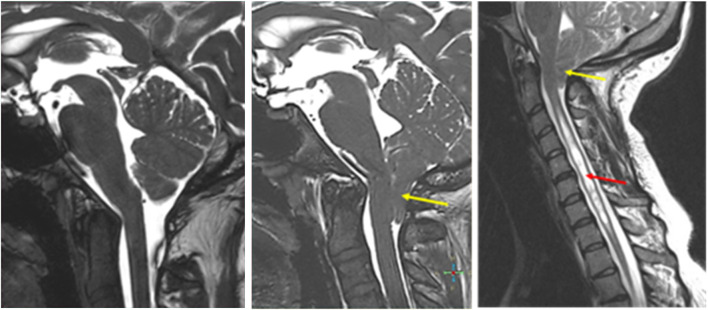
Midline sagittal MR images of a patent foramen magnum (left), tonsillar descent with obstruction of CSF flow (yellow arrow), and associated syringomyelia (red arrow).

The first image in Figure 11 below illustrates free CSF flow at the foramen magnum; the second image shows a Type 1 Chiari malformation. CSF flow is partially obstructed by the descent of the cerebellar tonsils. In some cases, tonsillar descent is compounded by CSF accumulation in the spinal cord, eventually causing syringomyelia (as shown in the third image), which further alters spinal CSF dynamics. This may or may not be associated with hydrocephalus. Degenerative disease affecting the caliber of the spinal canal (especially in the cervical and lumbar regions) is a common age-related change. An occult CSF leak (due to lumbar puncture, CNS trauma, or Ehlers–Danlos syndrome) can affect CSF circulation and be more difficult to localize (Mehta et al., 2024; Falatko et al., 2015).

Hemorrhages (which may affect CSF flow directly, or indirectly via blocking absorption) are described according to their CNS location or compartment, such as being intraventricular (IVH), subarachnoid (SAH), intracerebral (ICH), cerebellar, brainstem, spinal, subdural (SDH), and/or epidural (EDH). Multiple hemorrhages (or involvement of different compartments) may occur in the same patient, and a variety of underlying pathologies are possible, such as trauma, aneurysm, or arteriovenous malformation (Andrews and Engelhard, 2001; Engelhard, 2024; Sadeh et al., 2025). Common infections affecting CSF flow include meningitis, ventriculitis, abscess, encephalitis, and subdural empyema. A wide variety of organisms may be responsible. Tumors may also occur in a variety of locations, be focal or disseminated, and vary widely in histology, being primary or metastatic. In leptomeningeal metastases, tumor cells distribute themselves through CSF pathways (Engelhard, 2026). Other well-known conditions that entail alterations in CSF pathways or dynamics include pseudotumor cerebri, sinus thrombosis, skull base anomalies, and myelomeningocele (Naseri Kouzehgarani et al., 2021).

Representative diseases affecting the CSF circulation are listed by disease category in Table 6. This is just a general conceptual guide and cannot be all-inclusive. Note that diseases listed as generally having a “lesser effect” may still be significant enough to require treatment. In addition, some pathologies (such as traumatic brain injury) are listed in more than one spot on the table. There will always be a spectrum with respect to the clinical presentation of any given disease. Medications used to treat diseases (such as beta blockers) can diminish or possibly add to the effect of the disease. Of note is a recent report indicating that atrial fibrillation has a significant impact on CSF dynamics. A multimodal MRI study showed that patients with AF have reduced and aperiodic CSF flow compared with controls, with lower CSF flow volume and less rhythmicity to the flow patterns. This disruption could impair the function of the glymphatic system in clearing waste products, thus explaining why atrial fibrillation is associated with dementia (Hofer et al., 2025). If confirmed, this might prompt reconsideration of the clinical decision-making process regarding cardioversion of patents with atrial fibrillation. The presence of disease, while not necessarily producing an increased variability of CSF flow between affected individuals, certainly merits careful consideration of the different factors affecting CSF production, circulation, and absorption in that category of patient.

**TABLE 6:** Examples of diseases directly or indirectly affecting CSF circulation.

Disease category	CSF pathways directly obstructed		CSF pathways patent (impaired flow/Absorption; rare overproduction)	
	Major effect	Lesser effect	Major effect	Lesser effect
Congenital	Congenital hydrocephalus (e.g., aqueductal stenosis), congenital malformations, basilar invagination, Chiari malformation, and neural tube defects	Arachnoid cyst, tonsillar descent, Dandy–Walker malformation, syringomyelia, colloid cyst, and genetic syndromes	Communicating hydrocephalus, congenital heart disease, Ehlers–Danlos syndrome, and anatomical variations—septum pellucidum, and cisterns	Ciliary dyskinesia, developmental delay, mucopolysaccharidoses, leukodystrophy, and choroid plexus hyperplasia
Cardiorespiratory	Not expected	Not expected	Atrial fibrillation	Hypertension, congestive heart failure, severe sleep apnea, and pulmonary hypertension
Infectious	Bacterial meningitis, neurocysticercosis, ventriculitis, and post-infectious hydrocephalus	Tuberculous meningitis, mild post-infectious adhesions, granulomas, Lyme disease, and encephalitis	Communicating hydrocephalus (post-infection)	Viral meningitis and minor post-infectious changes
Neoplastic	Primary brain tumors, metastatic brain tumors, and leptomeningeal metastases	Primary brain tumors (e.g., low-grade glioma) and chronic lymphocytic infiltration	Choroid plexus papilloma	Elevated CSF protein, mild absorption deficit, and CNS lymphoma
Vascular	Intraventricular hemorrhage, subarachnoid hemorrhage, and post-hemorrhagic hydrocephalus	Intracerebral hemorrhage and stroke	Sinus thrombosis	Stroke and impaired venous drainage
Inflammatory	Neurosarcoidosis	Multiple sclerosis, chronic CNS inflammation, sarcoidosis, and rheumatoid arthritis	Multiple sclerosis and Sjögren’s syndrome	Chronic CNS inflammation, sarcoidosis, lupus erythematosus, and CNS vasculitis
Traumatic	Traumatic brain injury and post-traumatic hydrocephalus	Traumatic brain injury, syringomyelia, and subdural hematoma	Post-traumatic hydrocephalus and CSF leak	Chronic microvascular ischemia and disproportionately enlarged subarachnoid-space hydrocephalus (DESH)
Degenerative	Complete blockage of spinal canal	Spinal stenosis and spinal degenerative changes	Not expected	Alzheimer’s disease and other dementias
Idiopathic	Rare	Rare	Normal pressure hydrocephalus and idiopathic intracranial hypertension	Rare

Li et al., 2025; Katada et al., 2025; Nakajima et al., 2021; Jakimovski et al., 2020; Hashimoto et al., 2010; Jóhannsdóttir et al., 2024.

### 6.3 Neurodegenerative diseases

Neurodegenerative diseases are usually not associated with significant hydrocephalus or the impairment of CSF circulation. However, they may be associated with brain atrophy (pathological or as the result of aging), and this may facilitate delivery through the CSF. CSF clearance mechanisms may also be affected, which could prolong the dwell time of an agent administered into the CSF. Such diseases therefore deserve placement in a separate category. Diseases being considered for intraventricular gene therapy include Alzheimer’s disease, Parkinson’s disease, aromatic L-amino acid decarboxylase deficiency, sphingolipidoses (sphingolipid storage disease, Gaucher disease Types 1 and 2, GM1 gangliosidosis, GM2 gangliosidosis, Krabbe disease, and metachromatic leukodystrophy), mucopolysaccharidoses (MPS; Types I, II, IIIA, and IIIB), neuronal ceroid lipofuscinoses, spinal muscular atrophy, amyotrophic lateral sclerosis, leukodystrophies (adrenoleukodystrophy and Canavan disease), frontotemporal dementia, Huntington’s disease, Dravet syndrome, Rett syndrome, mesial temporal lobe epilepsy, and glutaric acidemia type 1 (Patel et al., 2024; Bey et al., 2020; García-González et al., 2024). The effect of many neurodegenerative diseases (and other diseases such as multiple sclerosis) on CSF flow is mediated by their impact upon local tissue volume—the production of brain atrophy (Jakimovski et al., 2020).

### 6.4 Effect of anesthesia on CSF flow

Patients requiring surgery for any reason (including Ommaya reservoir placement) would be expected to require anesthesia, although this may have very transient effects. As described in Section 5.3, anesthetized patients in recumbent positions are likely to have decreased motion and posture-mediated changes in CSF macrocirculation. Anesthetized rodents in a lateral sleep posture had higher CSF microcirculation than those in prone or supine postures (Lee et al., 2015). Differing anesthetics may increase or decrease respiratory rate, heart rate, blood pressure, cerebral blood flow, ICP, and cerebral vascular resistance (Slupe and Kirsch, 2018). Thus, either increased or decreased CSF flow rates could be produced, depending on the anesthetic agent. Propofol (Kahveci et al., 2001) and dexmedetomidine (Aryan et al., 2006) both decrease ICP and thus might increase and reroute the CSF microcirculation. Other anesthetics such as sevoflurane have reduced or no effect on CSF flow, or they decrease it (Zimmermann et al., 2025; Artru et al., 1997). One set of experiments using dexmedetomidine and isoflurane reported increased CSF microcirculation in rats (Benveniste et al., 2017), another using isoflurane, ketamine, and xylazine reported decreased CSF microcirculation in mice (Gakuba et al., 2018). Pentobarbital and halothane have been found to decrease the frequency of cilia beats, and halothane’s effect has continued for over an hour after the exposure ended (O’Callaghan and Sikand, 2012). Therefore, the effects of anesthesia may be a factor when therapeutic agents are being administered into the ventricle, as is usually the case in animal studies.

### 6.5 Direct assessment of the movement of therapeutic agents through the CSF

The most direct way to test the patency of CSF pathways and assess multiple sources of CSF flow variability would be by labeling and tracking the drug, macromolecule, or vector to be delivered. As mentioned above and in Supplementary Material 1, this has long been performed with radionuclide tracers, iodine, and gadolinium, both in animals and patients. More recently, antibodies and AAV9 capsids (as examples) can be labeled with PET isotopes such as carbon-11, fluorine-18, and zirconium-89, enabling noninvasive tracking and quantitative biodistribution studies *in vivo*, including after intraventricular administration (Pandit-Taskar et al., 2019; Allen et al., 2022; Bansal et al., 2025). Magnetic particle imaging (MPI) is a newer technique that is highly sensitive and can be applied to tracer studies using drugs, antibodies, or even cells (Fuller et al., 2019; Chandrasekharan et al., 2021; Ahlborg et al., 2025).

Given the considerable inter-patient variations in the circulation and clearance of therapeutic agents from the CSF and the effects of the disease process itself, a personalized tracer assessment could become a useful adjunct for an expanded set of conditions potentially amenable to intraventricular therapy, such as neurodegenerative diseases and not just leptomeningeal metastases (Hovd et al., 2022). Among noninvasive methods, a simultaneous, quantitative assessment of cardiac-related CSF dynamics throughout the ventricular system has now been renamed “4D flow MRI” (Vikner et al., 2024). The delivery and imaging technologies exist; improved clinical outcomes now need to be demonstrated for notoriously difficult diseases, while at the same time maintaining patient safety as the top priority.

### 6.6 Actionable guidance for intraventricular dosing in preclinical studies and clinical trials

The appreciation of these effects on CSF circulation will help predict variability in the intraventricular delivery of viral vectors and other biopharmaceuticals and inform the design of preclinical studies and clinical trials. Intraventricular dosing into the non-human primate (or rodent) brain is a significant technical challenge due to the small target area. It is critical that the catheter used for dosing remains within the ventricle and not be dislodged by movement of the brain relative to the catheter. Pressures within the dosing apparatus (e.g., Ommaya reservoir) must remain optimal to prevent the drug being inadvertent forced into adjacent parenchyma or tracking along the catheter. Published literature based on intraventricular dosing in laboratory species should include detailed descriptions of the methods to ensure that technical errors in the dosing procedure do not affect the results (Naseri Kouzehgarani et al., 2021).

In human studies, there may be significant variations among individuals according to age and disease and even within the same individual, depending on factors as seemingly minor as position or time of day. Dosing schedules need to take significant factors into consideration, just as weight is routinely kept in mind for pediatric drug dosing regimens. Actionable items for reflection in clinical trials and ensuring dose optimization are compiled in Table 7. As with most aspects of medicine, each patient requires an individualized assessment. There are pros and cons for using PC-MRI for determining, for instance, patient eligibility, but it could be a decisive diagnostic study for determining appropriate candidates for a clinical trial or helping to optimize an established therapy. A discussion of the physical properties and PK profile of the drug product, while mentioned in the table, are beyond the scope of this review.

**TABLE 7:** Considerations for clinical study recruiting and dose optimization.

Consideration	Example
Disease being treated	Leptomeningeal disease, Alzheimer’s, Huntington’s, ALS, and spinal cord injury
Exclusion criteria	Insurmountable obstructions: aqueductal stenosis and neoplastic obliteration of SAS
Imaging techniques	Complete MRI of CNS; PC-MRI—can be decisive for inclusion/exclusion; radionuclide ventriculography/cisternography
	Size of ventricles and SAS, even anatomy of the cisterns, will vary among individuals. CSF volume consideration
Effects on CSF production	Medications, abnormal laboratory values, and vital signs
Effects of age, youth vs. adult	Anatomical and physiological changes: pediatric vs. adult
	Ventricles, brain SAS, and spinal canal
Effect of age, older adult	Impact on ventricles (atrophy), brain SAS, and cervical spinal canal (degenerative stenosis)
Comorbid conditions	Any condition affecting CSF production and circulation (e.g., occult CSF leak and convexity adhesions post bleeding and infection) Disease-related anatomical or physiological changes
Physical properties of the drug product	Hydrophilic vs. hydrophobic, size, ionization, solubility, protein binding, and pathway adherence
CSF PK profile of the drug product	Immunogenicity/neutralization, distribution, clearance, and toxicity

Abbreviations: ALS - Amyotrophic lateral sclerosis, SAS - Spinal subarachnoid space, PK - pharmacokinetics. Other abbreviations are standard or already defined, such as MRI, CSF, PC-MRI.

In the past, intraventricular injections were thought to be vulnerable to bulk flow from the ventricles to the SAS, with rapid clearance of injected therapies (Pardridge, 2011; Noguchi et al., 2017). Rapid clearance would result in the need for frequent dosing (or chronic infusion), which would affect patient compliance and tolerance of the treatment (Yue and Shen, 2023). More recently, it has been reported that AAV viral particles administered via intraventricular delivery are transported throughout the brain, with an expression profile comparable to intracisternal injection (Hunter et al., 2025). Newer drug formulations have been developed that are more potent and therefore require a smaller dose (Krishnamurthy et al., 2024). Proteins and particles which adhere better to the ependymal surface should improve the delivery of therapeutic agents to targeted CNS regions (Zhang et al., 2022).

While systemic drug exposure would be expected to be less with intraventricular administration than intravenous delivery, serum levels could be used in addition to tracer or particle imaging to monitor therapy. Examinations prior to treatment should screen for any potential red flags, such as a change in blood pressure or medications, catheter obstruction, or disease progression. Awareness of the causes of the variability in the circulation of CSF and anticipating their consequences wherever appropriate will help ensure effective intraventricular drug delivery.

## 7 Conclusion

CNS fluid dynamics vary not only between species but within healthy individuals, depending on predictable circumstances, and from patient to patient, depending on their age and extent of disease. Beyond the essential contributions of 1) CSF production, 2) CSF pathway anatomy, 3) ICP, and 4) the cardiac and respiratory cycles, other physiological states such as timing within the circadian cycle, sleep, body position, ciliary motility, and even anesthesia cause variability in the circulation of CSF. One hypothesis about why animals yawn is that it is to increase the flow of CSF (Walusinski, 2014). MRI technology has enhanced our understanding of the continuous, dynamic movement of CSF between the intracranial ventricular compartments and the spinal and cranial SAS. Advancing age generally opens CSF pathways due to atrophic changes of the brain parenchyma. Within the spine, age-related degenerative changes generally restrict CSF flow. Pathology further impacts CSF dynamics, especially when directly obstructive in nature (e.g., leptomeningeal metastases) or when CSF absorption is impaired (e.g., meningitis or subarachnoid hemorrhage).

The dosage and scheduling of drugs given via the intraventricular route need to be carefully chosen in a given animal model and be even more precisely tailored according to individual patient characteristics. Challenges remain, including procedural risks (such as infection, bleeding, or neurological damage), concerns with drug distribution and clearance, and the risks of immunogenicity and/or neurotoxicity, including long-term effects (Naseri Kouzehgarani et al., 2021; Schreiner et al., 2025; Lee G.Y. et al., 2025). With a better understanding of the many factors that influence CSF circulation will come an improved ability to harness CSF pathways to deliver therapeutic drugs, macromolecules, and vectors to desired regions of the CNS.
