# Supplementary material for: Variability in the circulation of cerebrospinal fluid: causes and clinical implications for intraventricular drug delivery
Source: Front Drug Deliv. 2026 May 12;6:1735474. doi: 10.3389/fddev.2026.1735474 (PMC13201979; doi:10.3389/fddev.2026.1735474)
Supplement: Supplementary file 1 [file DataSheet2.pdf]

Supplement 2

Pediatric considerations in CSF flow dynamics

Differences between the child and adult patient populations which affect the circulation of CSF include: 1) allometric scaling, 2) differing skull anatomy, 3) developmental changes, 4) differences in CSF production, and 5) cardiac and pulmonary physiological differences. Brain and ventricular size have well established age-dependencies. [1] The most rapid growth in cortical and subcortical gray matter occurs at 0.5 years, and in white matter at 2.4 years. [2] In contrast, the ventricles only increase in size slightly during childhood but then enlarge at ever-increasing rates throughout life. Intra-CSF drug delivery needs to consider definite pharmacokinetic effects dependent on the ventricular volumes, the size of the brain regions being targeted, and how to address potential mixing in the ventricles, and adhesion to surfaces lining the pathways, before macromolecules or viral vectors will successfully arrive at the target tissues.

Regarding skull anatomy, the cranial bones of the fetus and newborn have unfused sutures which facilitate parturition. Fontanels are closed by 15-16 months (anterior fontanel) and 13-18 months (posterior fontanel) in approximately 50% of children, and closed by 24 months in approximately 90% of children. [3,4] The highly compliant skulls of infants with unfused sutures are correlated with substantially lower CSF macrocirculation. The flow velocity at the skull base of infants nearly doubles after suture closure, changing from 7.5 mm/s to 14.9 mm/s. [5] The individual's stage of development has a variety of effects on CSF circulation, and therefore the intraventricular administration of therapeutic agents. Different production rates of CSF create

pharmacokinetics that change relative to age. In adults, CSF production is reported at 18-24 mL/h. [6,7] In children ages 0-16 years, CSF production follows a logarithmic pattern, as expressed by the equation below. [8] Using this equation, it can be seen that 90% of the relative change in CSF production will occur by 9.0 years.

$$production\ \left(\frac{ml}{hr}\right) = 4.007 \times log_{10}(age) + 7.088$$

Many well-established developmental changes in the cardiopulmonary system also alter CSF dynamics. Two key age-dependent changes include decreases in heart rate and respiration rate. [9] Age-dependent trends in both are established, and equations for their median centiles are derived as seen below. Approximately 90% of the relative change in heart rate and respiration rate occur by 7.5 and 11.9 years, respectively.

$$heart\ rate\ (beats\ per\ minute) = 66.8 \times exp(-0.194 \times\ age) + 72.6$$

$$respiration\ rate\ (breaths\ per\ minute) = 36.4 \times exp(-0.307 \times\ age) + 16$$

So major age-related changes can be expected to taper due to fontanel closure (2 years), cardiac effects (7.5 years), CSF production (9 years), and pulmonary maturation (11.9 years). However, within these age ranges, considerations should be made for the age-dependent effects of skull stiffness, CSF production, and CSF macro-circulatory flow. After 12 years of age, considerations include the life-long enlargements of the ventricles and SAS, and a multitude of age-related changes in CSF production and clearance. [10]

References

1. Borzage M, Blüml S, Seri I. Equations to describe brain size across the continuum of human lifespan. *Brain Struct Funct*. 2014;219(1):141-150. doi:10.1007/s00429-012-0490-6
2. Bethlehem RAI, Seidlitz J, White SR, et al. Brain charts for the human lifespan. *Nature*. 2022;604(7906):525-533. doi:10.1038/s41586-022-04554-y
3. Pindrik J, Ye X, Ji BG, Pendleton C, Ahn ES. Anterior Fontanelle Closure and Size in Full-Term Children Based on Head Computed Tomography. *Clin Pediatr (Phila)*. 2014;53(12):1149-1157. doi:10.1177/0009922814538492
4. Kirkpatrick J, Bowie S, Mirjalili SA. Closure of the anterior and posterior fontanelle in the New Zealand population: A computed tomography study. *J Paediatrics Child Health*. 2019;55(5):588-593. doi:10.1111/jpc.14253
5. Wachi A, Kudo S, Sato K. Characteristics of cerebrospinal fluid circulation in infants as detected with MR velocity imaging. *Child's Nerv Syst*. 1995;11(4):227-230. doi:10.1007/BF00277658
6. Brinker T, Stopa E, Morrison J, Klinge P. A new look at cerebrospinal fluid circulation. *Fluids Barriers CNS*. 2014;11(1):10. doi:10.1186/2045-8118-11-10
7. Rubin RC, Henderson ES, Ommaya AK, Walker MD, Rall DP. The Production of Cerebrospinal Fluid in Man and Its Modification by Acetazolamide. *Journal of Neurosurgery*. 1966;25(4):430-436. doi:10.3171/jns.1966.25.4.0430
8. Yasuda T, Tomita T, McLone DG, Donovan M. Measurement of Cerebrospinal Fluid Output through External Ventricular Drainage in One Hundred Infants and Children: Correlation with Cerebrospinal Fluid Production. *Pediatr Neurosurg*. 2002;36(1):22-28. doi:10.1159/000048344
9. Fleming S, Thompson M, Stevens R, et al. Normal ranges of heart rate and respiratory rate in children from birth to 18 years of age: a systematic review of observational studies. *The Lancet*. 2011;377(9770):1011-1018. doi:10.1016/S0140-6736(10)62226-X
10. Mestre H, Tithof J, Du T, et al. Flow of cerebrospinal fluid is driven by arterial pulsations and is reduced in hypertension. *Nat Commun*. 2018;9(1):4878. doi:10.1038/s41467-018-07318-3
