# Supplementary material for: Variability in the circulation of cerebrospinal fluid: causes and clinical implications for intraventricular drug delivery
Source: Front Drug Deliv. 2026 May 12;6:1735474. doi: 10.3389/fddev.2026.1735474 (PMC13201979; doi:10.3389/fddev.2026.1735474)
Supplement: Supplementary file 2 [file DataSheet1.docx]

**Supplement 1**

Understanding CSF flow – historical aspects

As early as 1886, Knoll uncovered that pressure fluctuations in the cisterna magna of the rabbit changed not only with the heartbeat, but also with respiration. [1] In 1992, Schroth and Klose [2] were first to show with MRI, that CSF pulsations around the spine were the superposition of cardiac and respiratory cycles, the latter extending over several seconds. While the technique recorded CSF movement through a spinal MRI slice over an extended period, it was not quantitative. With the development of quantitative methods, it was confirmed that the changes in cerebral blood flow and pressure are the primary oscillatory drivers of the CSF macrocirculation. [3,4]

Prior to the development of computerized imaging *in vivo*, the anatomy of the CSF pathways, and conceptualization of how CSF was produced and circulated, were understood in general terms, based on cadaveric studies. In the 20^th^ Century, air or iodine contrast could be injected into the ventricular system and x-rays performed, including tomographic (*i.e.* planar) views, to provide enhanced anatomic detail. Radioactive tracers (radionuclides) were used next, with imaging performed by scintigraphy. [5,6] The advent of computerized axial tomography and then MRI greatly enhanced the ability of clinicians to understand the variations in CSF pathways between patients, and how disease processes could affect the circulation of CSF. Positron emission tomography (PET) has primarily been used to provide metabolic information within the CNS, but can be potentially used to localize tracers within CSF pathways.

The potential of MRI to quantify fluid velocities (via proton imaging) in humans was first suggested by Moran *et al.* in 1985, and confirmed by Feinberg and colleagues in 1987. [7,8]. The newer development of Phase Contrast - MRI (PC-MRI) led to breakthroughs in the understanding of cerebral blood flow, and the driving forces of CSF flow in humans. The early milestones in PC-MRI findings related to the CSF circulation are given in **Table S1-1**. Balédent and colleagues measured CSF oscillations in the spinal canal and cerebral aqueduct, and demonstrated that oscillations in the aqueduct (under normal physiological conditions) represent only about 10% of the oscillating volume, with 90% of the CSF oscillating in the intracranial SAS. [9]

**Table S1-1.** Early milestones in PC-MRI findings related to the CSF circulation

| **Year** | **Lead Author** | **Key Concept / Discovery** |
| --- | --- | --- |
| 1987 | Greitz | Introduced the concept of CSF oscillations resulting from arterial and venous blood flow interactions |
| 1991 | Enzmann | Found that CSF oscillation amplitudes differ in spinal canal vs. cerebral aqueduct |
| 1994 | Gideon | Showed that PC-MRI measurement of CSF oscillations aids in NPH diagnosis |
| 1996 | Bradley | Supported the clinical utility of PC-MRI for CSF oscillations in NPH diagnosis |
| 2000 | Alperin | Proposed ICP calculation from CSF and blood flow via numerical simulations |
| 2000 | Bateman | Highlighted vascular compliance influence on CSF oscillations |
| 2001 | Balédent | Characterized CSF flow waveform amplitude and timing versus cardiac cycle |

**Abbreviations:** NPH: normal pressure hydrocephalus. **References**: [9-15]

In the 1990s, 2D CINE PC-MRI was applied to the study of CSF oscillations in the cranio-spinal compartment and how CSF oscillations interact with cerebral blood flow. [16] This period marked the beginning of a new, open perspective on the dynamic physiology of the brain. It was understood that brain fluid dynamics cannot be decoupled from the broader cranio-spinal system, with the foramen magnum connecting the two subsystems. With the spinal compartment being not as rigid as the compartment encased within the skull, it was realized that the spinal CSF compartment serves to dampen ICP waves arising from the cranial CSF outflow.

Taken alone, aqueductal CSF dynamics do not entirely or always accurately reflect the alterations of the cranial spinal system. Aqueductal studies should be complemented by an assessment of cerebral vascular dynamics and CSF oscillations in intracranial and spinal SAS. Another factor coming into play is flow resistance in the aqueduct and its pressure gradient. Discoveries from the use of 2D CINE PC-MRI for the evaluation of intracranial flow are summarized in **Table S1-2**. In current PC-MRI protocols, spatial resolution is in the range of 0.5 – 1.0 mm, and image acquisition takes 2-5 minutes.

**Table S1-2.** Discoveries from the use of 2D CINE PC-MRI

| **Aspect** | **Measurement Approach** | **Typical Site(s)** | **Key Notes** |
| --- | --- | --- | --- |
| Intracranial Input | Sum of arterial flows | High cervical (C2-C3) | Arterial blood flow is dynamic, measured in both ICAs and vertebral/basilar arteries. |
| Intracranial Output | Venous system flows | High cervical (C2-C3) | Venous drainage is complex. It includes major sinuses, jugular veins, and untraceable small veins. PC-MRI cannot capture all venous paths. |
| CSF Oscillations | CSF flow measurement | Cerebral aqueduct | Assesses oscillatory movement between third and fourth ventricles. The aqueduct is the most common measurement site, with laminar flow occurring. |

**Abbreviations:** C: cervical, ICAs: internal carotid arteries

By coupling PC-MRI with echo planar imaging, it was possible to create new acquisition sequences capable of producing a fluid velocity map over the course of minutes without any cardiac or respiratory gating, and with a temporal resolution of 100 ms. This opened the way to study how cardiac and respiratory cycles jointly influence CSF (and blood) flow. The changes in intrathoracic volume and hydrostatic pressure due to breathing were then confirmed to be another important oscillatory driver of the CSF macrocirculation. Oscillations in CSF flow (both in frequency and in pressure) have now been quantitatively related to both cardiac and respiratory variability. [17].

The pattern of breathing was then found to further modify the effect on the CSF macrocirculation. Deep breathing and inspiration are both particularly strong drivers of CSF macrocirculation. [3,4] Mild respiratory support (such as continuous positive airway pressure [CPAP]) decreases the CSF stroke volume and amplitude. [18]. These observations have prompted numerous papers highlighting the positive importance of breathing on the CSF circulation. Ozturk *et al.* [19] showed that respiration can positively influence CSF flow in the brain, and suggested that CPAP might have therapeutic benefits for supporting glymphatic-lymphatic function in clearing waste from the CNS interstitium. Burman and Alperin [20] showed that breathing modulates CSF oscillations through changes in the venous outflow. The amount of waste products being transferred to the spinal canal (for elimination) during each respiratory cycle has been found to be significantly increased during slow and deeper abdominal breathing.

Glymphatic flow around the optic nerve and perivascular spaces is not amenable to imaging by PC-MRI because the velocities are far too low and these spaces are very small, creating conditions with small voxels, high partial-volume averaging, and low signal - hence leading to very low signal to noise ratios that make the use of standard PC-MRI ineffective. A summary of recent PC-MRI findings, especially related to respiration, is given in **Table S1-3.**

**Table S1-3.** Synopsis of later PC-MRI findings related to CSF flow

| **Study (Year)** | **Method** | **Main Findings** | **Reference (DOI)** |
| --- | --- | --- | --- |
| Klose et al. (2000)[21] | MRI | Caudally directed CSF flow in cerebral aqueduct peaks in late expiration; cranially directed flow peaks post-inspiration | 10.1002/(sici)1522-2586(200004)11:4<438::aid-jmri12>3.0.co;2-o |
| Dreha-Kulaczewski et al. (2015) [3] | MRI (forced breathing) | Inspiration is a more pronounced driving force of CSF flow than cardiac pulsation in humans | 10.1523/JNEUROSCI.3246-14.2015 |
| Chen et al. (2015) [22] | RT-PC MRI | Upward CSF movement into cranial cavity/lateral ventricles during inspiration; reversal during expiration | 10.1016/j.neuroimage.2015.07.073 |
| Daouk et al. (2017) [23] | MRI | Cerebral arterial, venous, and CSF flows correlate with respiration at cervical level | 10.1177/0284185116676655 |
| Yildiz et al. (2017) [24] | MRI | Respiratory and cardiac pulsations contribute to CSF velocity changes during deep breathing, but not natural breathing | 10.1002/jmri.25591 |
| Liu et al. (2024) [25] | RT-PC MRI | Under physiological breathing, CSF flow in cerebral aqueduct is more affected by respiratory cycles than flow in spinal canal | 10.1186/s12987-024-00520-0 |

*Deciphering Lundberg waves*

PC-MRI studies were undertaken to probe the nature of the enigmatic low-frequency ICP modulations called type A and B waves, discovered by Lundberg (in 1960) in patients with head injuries. [26] Lundberg type A waves are characterized by rapid and pronounced surges in ICP lasting from 5 to 20 minutes. They signal a reduction in brain compliance. Persistent Lundberg type A waves can be disruptive of CSF circulation and absorption. [27] Lundberg type B waves are also sharp and jagged, occurring every 1.5 to 2 minutes but presenting a lower amplitude than Lundberg type A waves. They may be indicative of a decrease in brain self-regulation or may simply be physiological in nature. Lundberg type C waves have also been described, which have an even smaller amplitude and occur 4-8 times per minute, but these are felt to have less clinical significance. It was reasonable to infer that Lundberg type A and B waves may have a significant impact on CSF flow, depending on the type of wave. Plots of CSF oscillations occurring over time, in relation to Lundberg A and B waves, are given in **Figure S1-1.** PC-MRI studies were able to clarify the nature of Lundberg waves, relating them to CSF flow oscillations, and the cardiac and respiratory cycles.


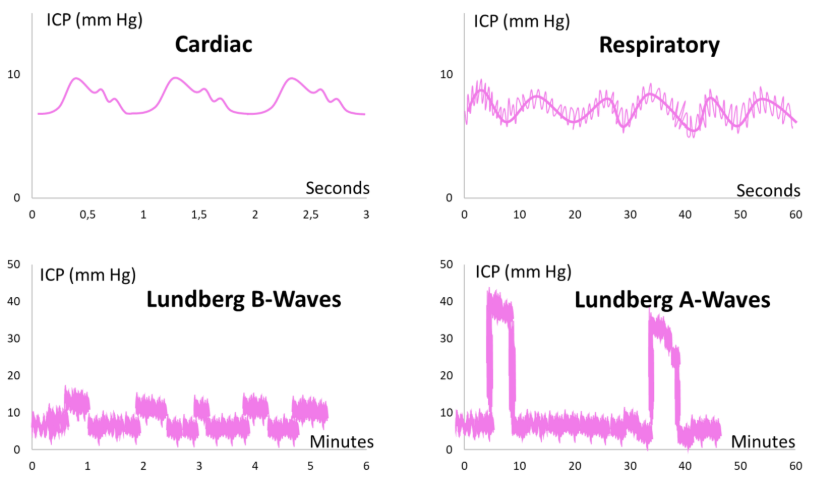


**Figure S1-1.** Factors influencing ICP over time and the types of CSF oscillations throughout the cranial spinal system. The x-axis varies the time period from three seconds to 60 minutes. The lower panels show the waves of increased ICP. The upper right panel, which is labelled “respiratory” is a combination of the cardiac and respiratory effects. Note that here, the heart rate is 60 beats per minute, and the respiratory rate is 6 per minute. B waves occur approximately every minute, and A waves are seen at a rate of 2 per hour. Source: Laboratory of Olivier Balédent.

**References**

1. Knoll P. Ueber die Druckschwankungen in der Cerebrospinalfluessigkeit und den Wechsel in der Blutfuelle des Centralen Nervensystems. *Sitzungsber Kaisefl Akad Wiss Wien Math Naturwiss Classe*. 1886:217-248.
2. Schroth G, Klose U. Cerebrospinal fluid flow: II. Physiology of respiration-related pulsations. *Neuroradiology*. 1992;35(1):10-15. doi:10.1007/BF00588271
3. Dreha-Kulaczewski S, Joseph AA, Merboldt KD, Ludwig HC, Gärtner J, Frahm J. Inspiration is the major regulator of human CSF flow. *J Neurosci*. 2015;35(6):2485-2491. doi:10.1523/JNEUROSCI.3246-14.2015
4. Kollmeier JM, Gürbüz-Reiss L, Sahoo P, et al. Deep breathing couples CSF and venous flow dynamics. *Sci Rep*. 2022;12(1):2568. doi:10.1038/s41598-022-06361-x
5. Proulx ST. Cerebrospinal fluid outflow: a review of the historical and contemporary evidence for arachnoid villi, perineural routes, and dural lymphatics. *Cell Mol Life Sci*. 2021;78(6):2429-2457. doi:10.1007/s00018-020-03706-5
6. Dichiro G. Movement of the cerebrospinal fluid in human beings. *Nature*. 1964;204:290-291. doi:10.1038/204290a0
7. Moran PR, Moran RA, Karstaedt N. Verification and evaluation of internal flow and motion. True magnetic resonance imaging by the phase gradient modulation method. *Radiology*. 1985;154(2):433-441. doi:10.1148/radiology.154.2.3966130
8. Feinberg DA, Mark AS. Human brain motion and cerebrospinal fluid circulation demonstrated with MR velocity imaging. *Radiology*. 1987;163(3):793-799. doi:10.1148/radiology.163.3.3575734
9. Balédent O, Henry-Feugeas MC, Idy-Peretti I. Cerebrospinal fluid dynamics and relation with blood flow: a magnetic resonance study with semiautomated cerebrospinal fluid segmentation. *Invest Radiol*. 2001;36(7):368-377. doi:10.1097/00004424-200107000-00003
10. Greitz D, Franck A, Nordell B. On the pulsatile nature of intracranial and spinal CSF-circulation demonstrated by MR imaging. *Acta Radiol*. 1993;34(4):321-328.
11. Enzmann DR, Pelc NJ. Normal flow patterns of intracranial and spinal cerebrospinal fluid defined with phase-contrast cine MR imaging. *Radiology*. 1991;178(2):467-474. doi:10.1148/radiology.178.2.1987610
12. Gideon P, Stohlberg F, Thomsen C, Gjerris F, Sorensen PS, Henriksen O. Cerebrospinal fluid flow and production in patients with normal pressure hydrocephalus studied by MRI. *Neuroradiology*. 1994;36(3):210-215. doi:10.1007/BF00588133
13. Bradley WG, Scalzo D, Queralt J, Nitz WN, Atkinson DJ, Wong P. Normal-pressure hydrocephalus: evaluation with cerebrospinal fluid flow measurements at MR imaging. *Radiology*. 1996;198(2):523-529. doi:10.1148/radiology.198.2.8596861
14. Alperin NJ, Lee SH, Loth F, Raksin PB, Lichtor T. MR - intracranial pressure (ICP): A method to measure intracranial elastance and pressure noninvasively by means of MR imaging: baboon and human study. *Radiology*. 2000;217(3):877-885. doi:10.1148/radiology.217.3.r00dc42877
15. Bateman GA. Vascular compliance in normal pressure hydrocephalus. *AJNR Am J Neuroradiol*. 2000;21(9):1574-1585.
16. Naidich TP, Altman NR, Gonzalez-Arias SM. Phase contrast cine magnetic resonance imaging: normal cerebrospinal fluid oscillation and applications to hydrocephalus. *Neurosurg Clin N Am*. 1993;4(4):677-705.
17. Liu P, Owashi K, Monnier H, Metanbou S, Capel C, Balédent O. Validating the accuracy of real-time phase-contrast MRI and quantifying the effects of free breathing on cerebrospinal fluid dynamics. *Fluids Barriers CNS*. 2024;21(1):25.
18. Yiallourou TI, Schmid Daners M, Kurtcuoglu V, et al. Continuous positive airway pressure alters cranial blood flow and cerebrospinal fluid dynamics at the craniovertebral junction. *Interdisciplinary Neurosurgery*. 2015;2(3):152-159. doi:10.1016/j.inat.2015.06.004
19. Ozturk B, Koundal S, Al Bizri E, et al. Continuous positive airway pressure increases CSF flow and glymphatic transport. *JCI Insight*. 2023;8(12):e170270. doi:10.1172/jci.insight.170270
20. Burman R, Alperin N. CSF ‐to‐blood toxins clearance is modulated by breathing through cranio–spinal CSF oscillation. *Journal of Sleep Research*. 2024;33(1):e14029. doi:10.1111/jsr.14029
21. Klose U, Strik C, Kiefer C, Grodd W. Detection of a relation between respiration and CSF pulsation with an echoplanar technique. *J Magn Reson Imaging*. 2000;11(4):438-444. doi:10.1002/(SICI)1522-2586(200004)11:4%3C438::AID-JMRI12%3E3.0.CO;2-O
22. Chen L, Beckett A, Verma A, Feinberg DA. Dynamics of respiratory and cardiac CSF motion revealed with real-time simultaneous multi-slice EPI velocity phase contrast imaging. *NeuroImage*. 2015;122:281-287. doi:10.1016/j.neuroimage.2015.07.073
23. Daouk J, Bouzerar R, Baledent O. Heart rate and respiration influence on macroscopic blood and CSF flows. *Acta Radiol*. 2017;58(8):977-982. doi:10.1177/0284185116676655
24. Yildiz S, Thyagaraj S, Jin N, et al. Quantifying the influence of respiration and cardiac pulsations on cerebrospinal fluid dynamics using real‐time phase‐contrast MRI. *Magnetic Resonance Imaging*. 2017;46(2):431-439. doi:10.1002/jmri.25591
25. Liu P, Owashi K, Monnier H, Metanbou S, Capel C, Balédent O. Validating the accuracy of real-time phase-contrast MRI and quantifying the effects of free breathing on cerebrospinal fluid dynamics. *Fluids Barriers CNS*. 2024;21(1):25. doi:10.1186/s12987-024-00520-0
26. Lundberg N. Continuous recording and control of ventricular fluid pressure in neurosurgical practice. *Acta Psychiatr Scand Suppl*. 1960;36(149):1-193.
27. Wijdicks EFM. Lundberg and his waves. *Neurocrit Care*. 2019;31(3):546-549. doi:10.1007/s12028-019-00689-5
